# Hepatic deficiency of the pioneer transcription factor FoxA restricts hepatitis B virus biosynthesis by the developmental regulation of viral DNA methylation

**DOI:** 10.1371/journal.ppat.1006239

**Published:** 2017-02-24

**Authors:** Vanessa C. McFadden, Rasha E. Shalaby, Saira Iram, Claudia E. Oropeza, Jennifer A. Landolfi, Alexander V. Lyubimov, Mark Maienschein-Cline, Stefan J. Green, Klaus H. Kaestner, Alan McLachlan

**Affiliations:** 1 Department of Microbiology and Immunology College of Medicine University of Illinois at Chicago 909 South Wolcott Avenue Chicago, IL, United States of America; 2 Toxicology Research Laboratory Department of Pharmacology College of Medicine University of Illinois at Chicago Chicago, IL, United States of America; 3 Research Resources Center College of Medicine University of Illinois at Chicago 835 South Wolcott Avenue Chicago, IL, United States of America; 4 Department of Genetics University of Pennsylvania School of Medicine Philadelphia, PA, United States of America; University of California, San Diego, UNITED STATES

## Abstract

The FoxA family of pioneer transcription factors regulates hepatitis B virus (HBV) transcription, and hence viral replication. Hepatocyte-specific FoxA-deficiency in the HBV transgenic mouse model of chronic infection prevents the transcription of the viral DNA genome as a result of the failure of the developmentally controlled conversion of 5-methylcytosine residues to cytosine during postnatal hepatic maturation. These observations suggest that pioneer transcription factors such as FoxA, which mark genes for expression at subsequent developmental steps in the cellular differentiation program, mediate their effects by reversing the DNA methylation status of their target genes to permit their ensuing expression when the appropriate tissue-specific transcription factor combinations arise during development. Furthermore, as the FoxA-deficient HBV transgenic mice are viable, the specific developmental timing, abundance and isoform type of pioneer factor expression must permit all essential liver gene expression to occur at a level sufficient to support adequate liver function. This implies that pioneer transcription factors can recognize and mark their target genes in distinct developmental manners dependent upon, at least in part, the concentration and affinity of FoxA for its binding sites within enhancer and promoter regulatory sequence elements. This selective marking of cellular genes for expression by the FoxA pioneer factor compared to HBV may offer the opportunity for the specific silencing of HBV gene expression and hence the resolution of chronic HBV infections which are responsible for approximately one million deaths worldwide annually due to liver cirrhosis and hepatocellular carcinoma.

## Author summary

This study demonstrates the connection between FoxA expression and gene silencing by DNA methylation *in vivo* during liver maturation. Insufficient FoxA expression results in selective developmentally regulated hepatitis B virus (HBV) silencing by DNA methylation. To our knowledge, this is the first *in vivo* demonstration that pioneer factors such as FoxA function by mediating the developmental demethylation of their target genes, leading to their tissue specific gene expression. Furthermore, our results strongly imply that the marking of cellular target genes for subsequent transcription later in development is dependent upon the level and timing of FoxA expression plus its affinity for its target sequences within enhancer and promoter regions. Consequently, these findings suggest that the appropriate control of FoxA activity during development could lead to the transcriptional inactivation of nuclear HBV covalently closed circular DNA by methylation and hence resolution of chronic HBV infection. This represents a clinical goal that current therapies are unable to attain, and hence suggests a potential route to a cure for this chronic infection which kills approximately 1 million individuals annually.

## Introduction

Hepatitis B virus (HBV) is a small enveloped DNA virus that infects the hepatocytes of human and ape liver [1]. Infection is generally considered to be restricted to hepatocytes by the viral receptor(s) which appears to include the sodium taurocholate cotransporting polypeptide (NTCP) [2]. Upon infection, the viral capsid transports the HBV 3.2kbp partially double-stranded DNA genome to the nucleus where it is converted into covalently closed circular (CCC) DNA [1, 3]. HBV CCC DNA appears to be highly stable and is resistant to current antiviral therapies used for the treatment of chronic viral infections [4–8]. It serves as the template for the transcription of the HBV 3.5kb, 2.4kb, 2.1kb and 0.7kb transcripts which are translated into the viral gene products, hepatitis B e antigen (HBeAg), core antigen (HBcAg), reverse transcriptase/DNA polymerase, surface antigen (HBsAg) and X-gene product (HBxAg) [1]. The viral polymerase binds to the HBV 3.5kb pregenomic RNA and this ribonucleoprotein particle is subsequently encapsidated by the core antigen polypeptide [9, 10]. Inside this immature core particle, the viral pregenomic RNA is reverse transcribed into genomic DNA generating a mature core particle [11, 12]. These mature particles can cycle genomic DNA back to the nucleus to increase the pool of nuclear HBV CCC DNA or bind surface antigen within the endoplasmic reticulum (ER) membrane and bud into the lumen of the ER [13]. Virus particles are subsequently transported through the Golgi apparatus and secreted from the cell into the circulation by vesicle trafficking [1, 14, 15].

Viral tropism is also determined by the liver-specific expression of the HBV genomic DNA [16–18]. At birth in HBV transgenic mice, HBV DNA is not transcribed and hence replication is absent from neonatal hepatocytes [19]. As the hepatocytes complete their postnatal differentiation program, liver-enriched transcription factor abundance increases [20] activating HBV transcription and replication which reach their maximal levels around the time of weaning [19, 21]. A number of liver-enriched transcription factors including the nuclear receptors, hepatocyte nuclear factor 4 (HNF4), retinoid X receptor (RXR), peroxisome proliferator-activated receptor (PPAR), farnesoid X receptor (FXR), liver receptor homolog 1 (LRH1), estrogen related receptor (ERR), the homeobox factor, hepatocyte nuclear factor 1 (HNF1), the basic-leucine zipper factors, CCAAT-enhancer-binding proteins (C/EBP) and the winged-helix transcription factors, forkhead box protein A/hepatocyte nuclear factor 3 (FoxA/HNF3) bind to the HBV enhancer and promoter regulatory sequence elements [17, 22, 23]. The nuclear receptors governing HBV transcription have been shown to be a major determinant of viral tropism [16, 24]. In contrast, the importance of the other liver-enriched transcription factors in the regulation of HBV transcription and replication is unclear [16]. Interestingly, the FoxA proteins are pioneer transcription factors which bind to and mark cellular genes for expression at later stages during development [25–28]. In particular, FoxA has been shown to bind and mark the albumin enhancer and α-fetoprotein promoter for subsequent gene expression during liver development and definitive endoderm differentiation, respectively [25, 29, 30]. As HBV transcription is developmentally restricted with detectable viral biosynthesis occurring shortly after birth in the neonatal liver [19, 21], it was of interest to determine the importance of FoxA in this process and whether its role as a pioneer transcription factor contributed to HBV RNA synthesis during hepatocyte maturation.

Pioneer transcription factors bind to their target genes at an early stage in tissue development and modulate chromatin structure permitting gene expression at a later developmental stage [25, 26, 29]. Subsequent binding of additional transcription factors to these accessible enhancer and promoter regulatory sequence elements leads to the recruitment of coactivators, the mediator complex, the general transcription factors and RNA polymerase II generating a functional preinitiation complex which directs transcription initiation and gene expression [29, 31, 32]. In tissues that do not express the required pioneer factor, the target genes for these factors are not marked early in development and may never be expressed in these tissues presumably due to a generally repressive chromatin environment. The mechanism(s) of tissue specific silencing of pioneer factor target genes in the tissues that do not express these factors is poorly defined. However, it appears that DNA methylation of cytosine residues to 5-methylcytosine may contribute to this process as the regulatory regions of these genes are often hypomethylated in cells and tissues where they are or may be expressed and hypermethylated in tissues where these genes are silent [33–38]. Direct evidence for a link between pioneer factor control of tissue-specific developmental gene expression and regulatory region methylation status is limited [36, 37] as deletion of pioneer factors such as FoxA leads to the loss of the gene expression profile essential for normal tissue development and hence is generally lethal [26].

In this study, the effect of FoxA-deficiency on the liver-specific expression of the viral genome was investigated in the HBV transgenic mouse model of chronic infection [21]. FoxA-deficiency resulted in the loss of HBV transcription and replication, essentially leading to a “cure” of the chronic viral infection. The deficiency in FoxA was associated with the methylation of the HBV genomic DNA indicating directly that the developmental expression of the FoxA pioneer factors is essential to prevent the epigenetic silencing of the viral genome. Developmental studies demonstrated that HBV genomic DNA was fully methylated at birth when viral transcription is absent. This indicates that FoxA mediates, directly or indirectly, the postnatal demethylation of the viral DNA leading to its subsequent expression during the process of hepatocyte maturation. Additionally, the residual FoxA3 synthesis in these mice was sufficient to mark and support the expression of the set of hepatocyte-specific genes which are dependent upon this pioneer factor such that liver maturation occurred and viable animals were produced. This indicates that there are different developmental expression requirements for appropriately marking essential hepatocyte-specific cellular genes compared to HBV genomic DNA with this specific pioneer factor which ultimately can influence their subsequent expression during cellular differentiation. This may be a general mechanism of action of pioneer factors and suggests the concentration of the pioneer factor(s) combined with the affinity and number of binding sites in enhancer and promoter sequences may determine the stage of development and differentiation when tissue-specific genes are marked for subsequent expression. Failure to correctly mark genes for expression by the pioneer factor(s) may ultimately result in their silencing by DNA methylation.

## Results

The pioneer transcription factor, FoxA, has been shown to modulate HBV transcription and replication in cell culture [16, 25, 39–44]. Moreover, the enhanced expression of FoxA2 in the liver of HBV transgenic mice has been shown to inhibit viral biosynthesis whereas the absence of FoxA3 in this model system displayed only a modest effect on HBV transcription and replication under fasting conditions [45, 46]. Therefore it was of interest to more precisely define the *in vivo* importance of the FoxA transcription factors for HBV biosynthesis. Consequently, liver-specific FoxA2-null, liver-specific FoxA1-null:FoxA2-null and liver-specific FoxA1:FoxA2-null:FoxA3-heterozygous HBV transgenic mice, HBVFoxA2^fl/fl^AlbCre, HBVFoxA1^fl/fl^FoxA2^fl/fl^AlbCre and HBVFoxA1^fl/fl^FoxA2^fl/fl^FoxA3^+/-^AlbCre, respectively, were generated and characterized.

### Characterization of FoxA expression in HBVFoxA2^fl/fl^AlbCre, HBVFoxA1^fl/fl^FoxA2^fl/fl^AlbCre and HBVFoxA1^fl/fl^FoxA2^fl/fl^FoxA3^+/-^AlbCre HBV transgenic mice

**Fig 1:**
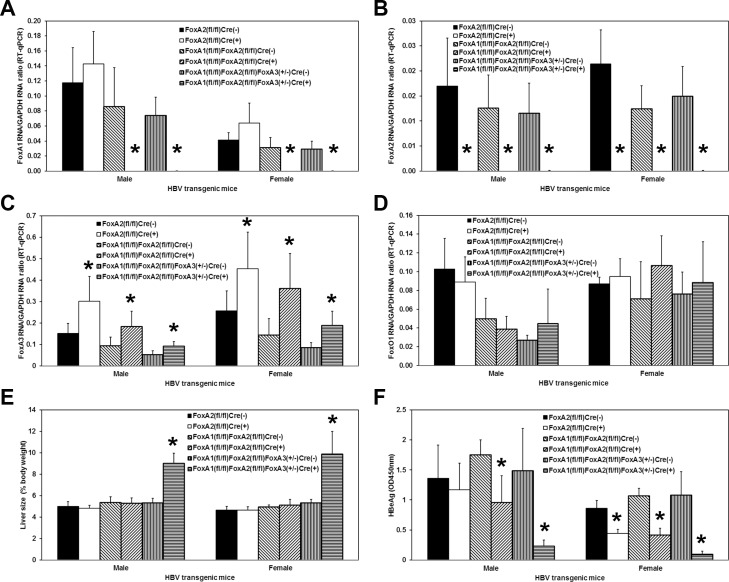
Effects of FoxA deletion on liver FoxA1, FoxA2, FoxA3 and FoxO1 transcript levels, liver size and serum HBeAg in adult HBV transgenic mice. Quantitative analysis of the (A) FoxA1, (B) FoxA2, (C) FoxA3 and (D) FoxO1 transcripts by RT-qPCR in the HBV transgenic mice. The GAPDH transcript was used as an internal control for the quantitation of the FoxA1, FoxA2, FoxA3 and FoxO1 RNAs. The mean relative FoxA1, FoxA2, FoxA3 and FoxO1 transcript levels plus standard deviations derived from male and female FoxA-expressing (HBVFoxA2^fl/fl^AlbCre(-), HBVFoxA1^fl/fl^FoxA2^fl/fl^AlbCre(-) and HBVFoxA1^fl/fl^FoxA2^fl/fl^FoxA3^+/-^AlbCre(-)) and FoxA-deleted (HBVFoxA2^fl/fl^AlbCre(+), HBVFoxA1^fl/fl^FoxA2^fl/fl^AlbCre(+) and HBVFoxA1^fl/fl^FoxA2^fl/fl^FoxA3^+/-^AlbCre(+)) HBV transgenic mice is shown. The levels of the transcripts which are statistically significantly different between Cre(-) and Cre(+) HBV transgenic mice by a Student’s t-test (p<0.05) are indicated with an asterisk (*). Similar quantitative analysis of (E) liver size and (F) serum HBeAg levels is also shown. Average number of mice per group was 6.5±1.9 (Range: 3–9).

HBVFoxA2^fl/fl^AlbCre(+), HBVFoxA1^fl/fl^FoxA2^fl/fl^AlbCre(+) and HBVFoxA1^fl/fl^FoxA2^fl/fl^FoxA3^+/-^AlbCre(+) transgenic mice were viable and displayed no overt phenotype [47]. In contrast, neonatal and adult HBVFoxA1^fl/fl^FoxA2^fl/fl^FoxA3^-/-^AlbCre(+) transgenic mice were not observed. The levels of the FoxA RNAs in the livers of the wildtype HBV transgenic mice (HBVFoxA2^fl/fl^AlbCre(-), HBVFoxA1^fl/fl^FoxA2^fl/fl^AlbCre(-) and HBVFoxA1^fl/fl^FoxA2^fl/fl^FoxA3^+/-^AlbCre(-)) were compared with the levels of the FoxA RNAs in the FoxA-deleted HBV transgenic mice (HBVFoxA2^fl/fl^AlbCre(+), HBVFoxA1^fl/fl^FoxA2^fl/fl^AlbCre(+) and HBVFoxA1^fl/fl^FoxA2^fl/fl^FoxA3^+/-^AlbCre(+)) (Fig 1A–1C). FoxA1 RNA was reduced by 300–1700 fold in the HBV transgenic mice with any FoxA1^fl/fl^AlbCre(+) genotype (Fig 1A). FoxA2 RNA was reduced by more than 200-fold in the HBV transgenic mice with any FoxA2^fl/fl^AlbCre(+) genotype (Fig 1B). FoxA3 RNA was increased by approximately 2-fold in all the HBV transgenic mice with any FoxA2^fl/fl^AlbCre(+) genotype indicating that the loss of FoxA2 expression resulted in a modest increase in FoxA3 expression (Fig 1B and 1C) presumably reflecting the coordinated transcriptional regulation of the FoxA factor abundances during development. In contrast, FoxO1 RNA levels although somewhat variable appeared to be relatively insensitive to changes in FoxA factor abundance (Fig 1D).

### Effect of FoxA deletion on liver size and serum HBeAg in HBV transgenic mice

As note previously for the FoxA1^fl/fl^FoxA2^fl/fl^AlbCre(+) mice [47], none of the mutant mice in this study showed significant changes in blood biochemistry, including levels of alkaline phosphatase, alanine aminotransferase (ALT), aspartate aminotransferase (AST), bilirubin, blood urea nitrogen, creatine, glucose, triglyceride, albumin (Alb), total protein (TP), globulin (TP-Alb), Alb:globulin ratio, and triacylglycerol, with the exception of cholesterol which was approximately 2-fold lower in the HBVFoxA1^fl/fl^FoxA2^fl/fl^FoxA3^+/-^AlbCre(+) mice (HBVFoxA1^fl/fl^FoxA2^fl/fl^FoxA3^+/-^AlbCre(-), 114±20 mg/dl; HBVFoxA1^fl/fl^FoxA2^fl/fl^FoxA3^+/-^AlbCre(+), 60±7 mg/dl, p = 0.01, n = 3), compared with the corresponding controls.

In contrast to the FoxA1^fl/fl^FoxA2^fl/fl^AlbCre(+) mice [47], the HBVFoxA1^fl/fl^FoxA2^fl/fl^FoxA3^+/-^AlbCre(+) mice displayed hepatomegaly (Fig 1E). The livers of these mice were approximately 1.7–1.9 fold larger than their AlbCre negative wildtype controls. The liver-specific FoxA2-null and FoxA1:FoxA2-null HBV transgenic mice (FoxA2^fl/fl^AlbCre(+) and FoxA1^fl/fl^FoxA2^fl/fl^AlbCre(+), respectively) displayed a relatively modest, 1.2–2.5 fold, reduction in the level of serum HBeAg (Fig 1F). However the HBVFoxA1^fl/fl^FoxA2^fl/fl^FoxA3^+/-^AlbCre(+) mice showed dramatically reduced levels of serum HBeAg, approximately 5–10 fold lower than their wildtype controls (Fig 1F). As HBeAg is translated from the HBV 3.5kb precore RNA [48], these observations suggest that the FoxA-deficiency in these HBVFoxA1^fl/fl^FoxA2^fl/fl^FoxA3^+/-^AlbCre(+) mice is associated with a marked decrease in the synthesis of the HBV 3.5kb precore RNA.

### Effect of FoxA deletion on viral transcription in HBV transgenic mice

**Fig 2:**
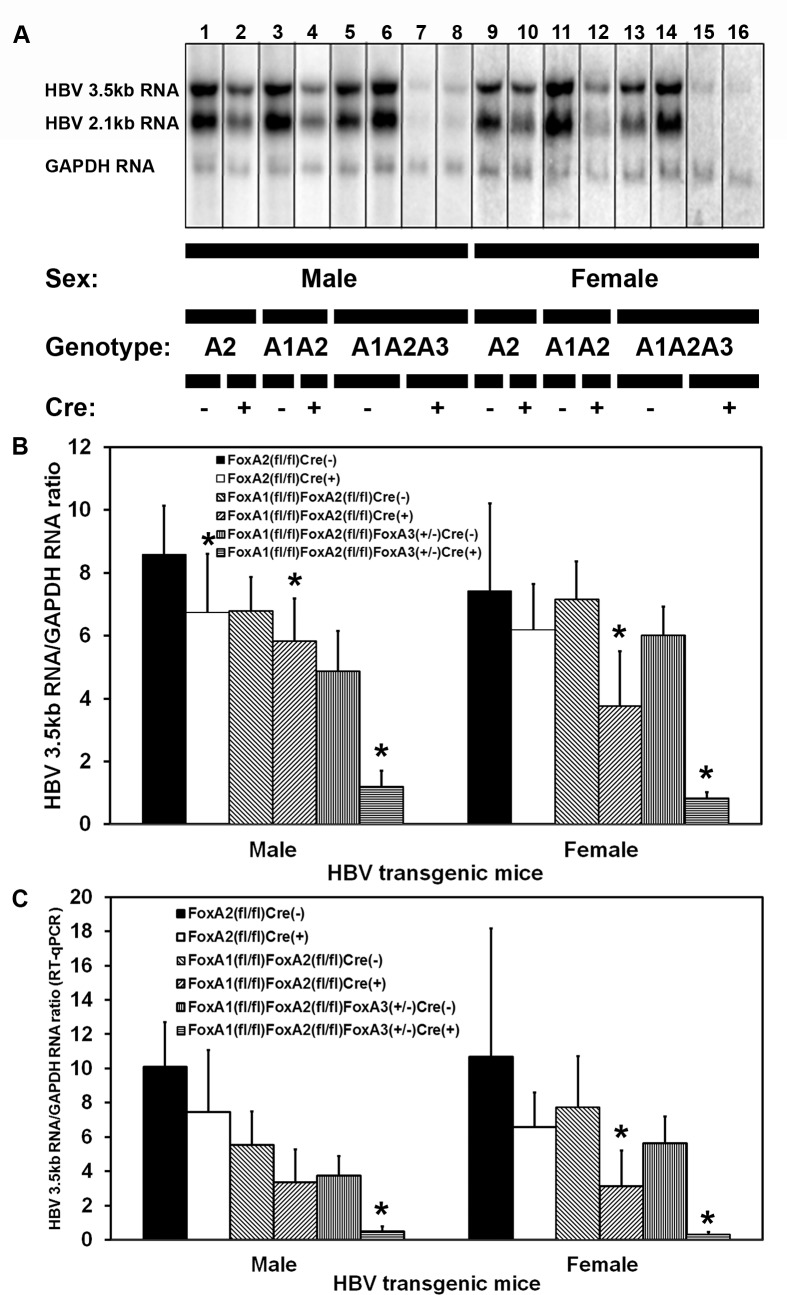
RNA (Northern) filter hybridization and RT-qPCR analysis of HBV transcripts in the livers of adult HBV transgenic mice. (A) RNA (Northern) filter hybridization analysis of representative mice of each sex and genotype are shown. Noncontiguous lanes from multiple analysis are presented. The probes used were HBV*ayw* genomic DNA plus GAPDH cDNA. FoxA-expressing (HBVFoxA2^fl/fl^AlbCre(-), HBVFoxA1^fl/fl^FoxA2^fl/fl^AlbCre(-) and HBVFoxA1^fl/fl^FoxA2^fl/fl^FoxA3^+/-^AlbCre(-)) and FoxA-deleted (HBVFoxA2^fl/fl^AlbCre(+), HBVFoxA1^fl/fl^FoxA2^fl/fl^AlbCre(+) and HBVFoxA1^fl/fl^FoxA2^fl/fl^FoxA3^+/-^AlbCre(+)) HBV transgenic mice are indicated (Genotype A2, A1A2 and A1A2A3, respectively). The glyceraldehyde 3-phosphate dehydrogenase (GAPDH) transcript was used as an internal control for the quantitation of the HBV 3.5kb RNA. (B) Quantitative analysis by RNA (Northern) filter hybridization of the HBV 3.5kb transcript in the HBV transgenic mice. The mean HBV 3.5kb transcript levels plus standard deviations are indicated. Average number of mice per group was 6.6±1.9 (Range: 4–9). The levels of the HBV 3.5kb transcript which are statistically significantly different between Cre(-) and Cre(+) HBV transgenic mice by a Student’s t-test (p<0.05) are indicated with an asterisk (*). (C) Quantitative analysis by RT-qPCR of the HBV 3.5kb transcript in the HBV transgenic mice. The mean HBV 3.5kb transcript levels plus standard deviations are indicated. Average number of mice per group was 6.5±1.8 (Range: 4–9). The levels of the HBV 3.5kb transcript which are statistically significantly different between Cre(-) and Cre(+) HBV transgenic mice by a Student’s t-test (p<0.05) are indicated with an asterisk (*).

HBV transgenic mice that lacked liver-specific expression of FoxA2 (HBVFoxA2^fl/fl^AlbCre(+)), FoxA1 plus FoxA2 (HBVFoxA1^fl/fl^FoxA2^fl/fl^AlbCre(+)), or FoxA1 plus FoxA2 with reduced levels of FoxA3 (HBVFoxA1^fl/fl^FoxA2^fl/fl^FoxA3^+/-^AlbCre(+)) were examined for their steady state levels of HBV transcripts by analysis of total liver RNA (Fig 2). The steady state levels of the HBV 3.5kb and 2.1kb transcripts in the livers of the HBV transgenic mice expressing only reduced levels of FoxA3, HBVFoxA1^fl/fl^FoxA2^fl/fl^FoxA3^+/-^AlbCre(+), were greatly diminished relative to the controls (Fig 2). The liver-specific deletion of FoxA2 or FoxA1 plus FoxA2 (HBVFoxA2^fl/fl^AlbCre(+) and HBVFoxA1^fl/fl^FoxA2^fl/fl^AlbCre(+) mice, respectively) reduced the levels of the HBV 3.5kb transcripts to a relatively modest extent, 1.4–2.5 fold, by RNA filter hybridization (Fig 2B) and RT-qPCR analysis (Fig 2C). These observations are consistent with the observed reduction in serum HBeAg (Fig 1F) and suggests that the lack of FoxA1 and FoxA2 modestly reduced the level of the HBV 3.5kb precore RNA that encodes the HBeAg [48]. HBV transgenic mice expressing only reduced levels of FoxA3, HBVFoxA1^fl/fl^FoxA2^fl/fl^FoxA3^+/-^AlbCre(+), displayed a 4–8 fold reduction in the HBV 3.5kb RNA by RNA filter hybridization analysis (Fig 2B) and a 8–16 fold reduction by RT-qPCR analysis (Fig 2C). These reductions in HBV 3.5kb RNA are consistent with the observe reduction in serum HBeAg seen in these FoxA-deficient mice, HBVFoxA1^fl/fl^FoxA2^fl/fl^FoxA3^+/-^AlbCre(+) (Fig 1F).

### Effect of FoxA deletion on viral replication intermediates in HBV transgenic mice

**Fig 3:**
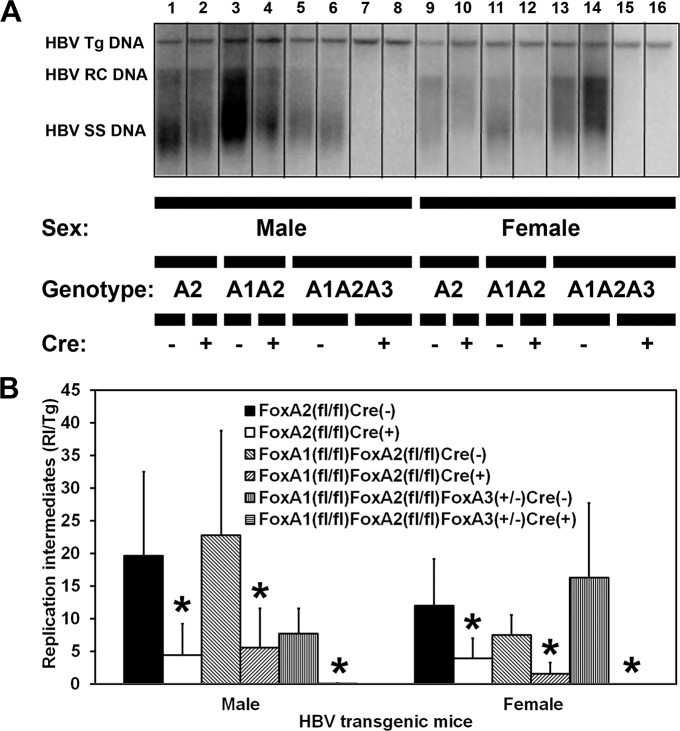
DNA (Southern) filter hybridization analysis of HBV DNA replication intermediates in the livers of adult HBV transgenic mice. (A) DNA (Southern) filter hybridization analysis of representative mice of each sex and genotype are shown. Noncontiguous lanes from multiple analysis are presented. The probe used was HBVayw genomic DNA. FoxA-expressing (HBVFoxA2^fl/fl^AlbCre(-), HBVFoxA1^fl/fl^FoxA2^fl/fl^AlbCre(-) and HBVFoxA1^fl/fl^FoxA2^fl/fl^FoxA3^+/-^AlbCre(-)) and FoxA-deleted (HBVFoxA2^fl/fl^AlbCre(+), HBVFoxA1^fl/fl^FoxA2^fl/fl^AlbCre(+) and HBVFoxA1^fl/fl^FoxA2^fl/fl^FoxA3^+/-^AlbCre(+)) HBV transgenic mice are indicated (Genotype A2, A1A2 and A1A2A3, respectively). The HBV transgene (Tg) was used as an internal control for the quantitation of the HBV replication intermediates. Tg = HBV transgene; RC = HBV relaxed circular replication intermediates; SS = HBV single stranded replication intermediates. (B) Quantitative analysis of the HBV DNA replication intermediate (RI) levels in HBV transgenic mice. The mean DNA replication intermediate levels plus standard deviations are indicated. Average number of mice per group was 6.7±1.8 (Range: 4–9). The levels of replication intermediates which are statistically significantly different between Cre(-) and Cre(+) HBV transgenic mice by a Student’s t-test (p<0.05) are indicated with an asterisk (*).

HBV transgenic mice that lacked liver-specific expression of FoxA2 (HBVFoxA2^fl/fl^AlbCre(+)), FoxA1 plus FoxA2 (HBVFoxA1^fl/fl^FoxA2^fl/fl^AlbCre(+)), or FoxA1 plus FoxA2 with reduced levels of FoxA3 (HBVFoxA1^fl/fl^FoxA2^fl/fl^FoxA3^+/-^AlbCre(+)) were examined for their steady state levels of HBV replication intermediates by analysis of total liver DNA (Fig 3). HBV DNA replication intermediates were undetectable in the livers of the HBV transgenic mice expressing only reduced levels of FoxA3 (HBVFoxA1^fl/fl^FoxA2^fl/fl^FoxA3^+/-^AlbCre(+)) (Fig 3). The liver-specific deletion of FoxA2 or FoxA1 plus FoxA2 (HBVFoxA2^fl/fl^AlbCre(+) and HBVFoxA1^fl/fl^FoxA2^fl/fl^AlbCre(+) mice, respectively) reduced the levels of the HBV replication intermediates by 3–5 fold by DNA filter hybridization (Fig 3B). These decreases in replication intermediates were slightly greater than those observed for the HBV 3.5kb RNA but they are consistent with previous findings which suggest that viral DNA synthesis can be sensitive to small changes in HBV transcription [4, 49, 50]. HBV transgenic mice expressing only reduced levels of FoxA3, HBVFoxA1^fl/fl^FoxA2^fl/fl^FoxA3^+/-^AlbCre(+), failed to displayed any detectable HBV replication intermediates (Fig 3B) despite expressing low levels of HBV 3.5kb RNA (Fig 2B and 2C). These observations suggest that either the level of RNA synthesis is insufficient to support HBV DNA synthesis in this model of chronic HBV infection or viral replication is being suppressed at both the transcriptional and posttranscriptional levels as has been suggested previously [51].

### Effect of FoxA deficiency on viral HBcAg distribution and cellular morphology within the livers of HBV transgenic mice

**Fig 4:**
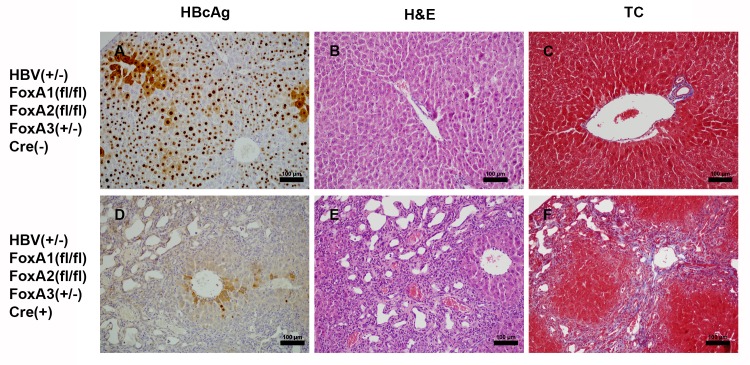
Histological analysis of liver samples from adult HBV transgenic mice. Control FoxA-expressing (HBVFoxA1^fl/fl^FoxA2^fl/fl^FoxA3^+/-^AlbCre(-), panels A-C) and FoxA-deleted (HBVFoxA1^fl/fl^FoxA2^fl/fl^FoxA3^+/-^AlbCre(+), panels D-F) HBV transgenic mice are indicated. Immunohistochemical staining indicates the presence of nuclear HBcAg throughout the liver whereas cytoplasmic staining is located primarily in the centrolobular hepatocytes in control mice (panel A) whereas HBcAg is minimally detectable in the FoxA-deleted mice (panel D). Hematoxylin and eosin (H&E) staining indicates biliary epithelial proliferation in the FoxA-deleted mice (panel E) which is absent in the control mice (panel B). Trichrome (TC) staining indicates bridging portal fibrosis in the FoxA-deleted mice (panel F) which is absent in the control mice (panel C). The black size bar is 100 μm.

**Fig 5:**
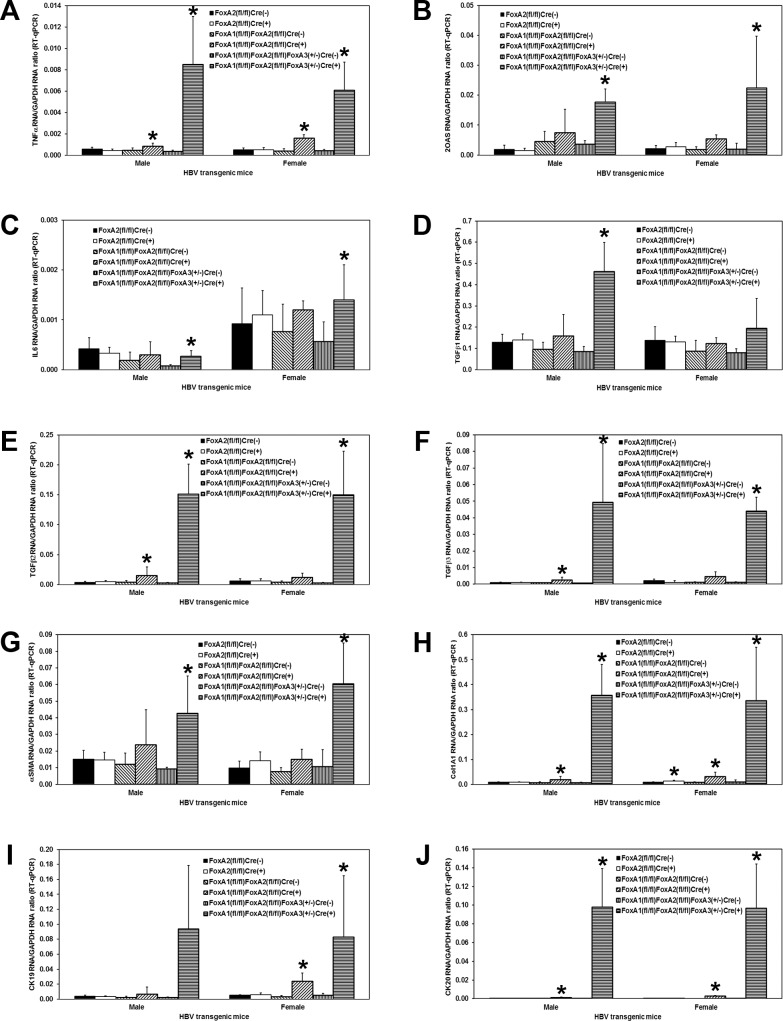
Effects of FoxA deletion on liver TNFα, 2OAS, IL6, TGFβ1, TGFβ2, TGFβ3, αSMA, Col1A1, CK19 and CK20 transcript levels in adult HBV transgenic mice. Quantitative analysis of the (A) TNFα, (B) 2OAS, (C) IL6, (D) TGFβ1, (E) TGFβ2, (F) TGFβ3, (G) αSMA, (H) Col1A1, (I) CK19 and (J) CK20 transcripts by RT-qPCR in the HBV transgenic mice. The GAPDH transcript was used as an internal control for the quantitation of the TNFα, 2OAS, IL6, TGFβ1, TGFβ2, TGFβ3, αSMA, Col1A1, CK19 and CK20 RNAs. The mean relative TNFα, 2OAS, IL6, TGFβ1, TGFβ2, TGFβ3, αSMA, Col1A1, CK19 and CK20 transcript levels plus standard deviations derived from male and female FoxA-expressing (HBVFoxA2^fl/fl^AlbCre(-), HBVFoxA1^fl/fl^FoxA2^fl/fl^AlbCre(-) and HBVFoxA1^fl/fl^FoxA2^fl/fl^FoxA3^+/-^AlbCre(-)) and FoxA-deleted (HBVFoxA2^fl/fl^AlbCre(+), HBVFoxA1^fl/fl^FoxA2^fl/fl^AlbCre(+) and HBVFoxA1^fl/fl^FoxA2^fl/fl^FoxA3^+/-^AlbCre(+)) HBV transgenic mice is shown. The levels of the transcripts which are statistically significantly different between Cre(-) and Cre(+) HBV transgenic mice by a Student’s t-test (p<0.05) are indicated with an asterisk (*). Average number of mice per group was 6.5±1.8 (Range: 4–9).

Immunohistochemical analysis of the livers of HBV transgenic mice expressing only reduced levels of FoxA3, HBVFoxA1^fl/fl^FoxA2^fl/fl^FoxA3^+/-^AlbCre(+), display greatly reduced HBcAg staining within the liver lobule (Fig 4D) compared to control mice (Fig 4A). Indeed, weak cytoplasmic and limited nuclear staining is observed in only a very limited number of hepatocytes located close to the central vein (Fig 4D). These findings are consistent with the reductions in HBV RNA and DNA synthesis observed in these mice (Figs 2 and 3). Previous histological analysis of FoxA1:FoxA2-null mice (HBVFoxA1^fl/fl^FoxA2^fl/fl^AlbCre(+)) had demonstrated that their livers displayed dilated, disorganized, and expanded bile ducts due to cholangiocyte proliferation which is characteristic of biliary hyperplasia [47]. Additionally, these proliferating bile ducts were surrounded by extracellular matrix which was comprised of collagen bundles which is typical of liver fibrosis [47]. Hematoxylin and eosin staining of livers from HBV transgenic mice expressing only reduced levels of FoxA3, HBVFoxA1^fl/fl^FoxA2^fl/fl^FoxA3^+/-^AlbCre(+), also displayed similar biliary hyperplasia which was more severe than that seen in the FoxA1:FoxA2-null mice, HBVFoxA1^fl/fl^FoxA2^fl/fl^AlbCre(+) (Figs 4B, 4E and 5). Similarly, the fibrosis observed by trichrome staining of the livers from the HBV transgenic mice expressing only reduced levels of FoxA3 (HBVFoxA1^fl/fl^FoxA2^fl/fl^FoxA3^+/-^AlbCre(+)) was more severe than that seen in the FoxA1:FoxA2-null mice (HBVFoxA1^fl/fl^FoxA2^fl/fl^AlbCre(+)), consistent with mild to moderate, bridging portal fibrosis (Figs 4C, 4F and 5). The extensive biliary epithelial cell proliferation and fibrosis alone, in the absence of any observable hepatocyte damage as measured by serum ALT and AST, appear to explain the noted increase in liver size associated with the HBVFoxA1^fl/fl^FoxA2^fl/fl^FoxA3^+/-^AlbCre(+) mouse (Fig 1E). No differences in liver tissue histology were apparent between HBV transgenic and non-transgenic mice of the same FoxA genotype indicating that HBV biosynthesis did not contribute to the observed liver phenotypes.

### Effect of FoxA deletion on cytokine production, stellate cell activation and biliary epithelial cell proliferation within the livers of HBV transgenic mice

Histological analysis of the livers of HBV transgenic mice expressing only reduced levels of FoxA3, HBVFoxA1^fl/fl^FoxA2^fl/fl^FoxA3^+/-^AlbCre(+), indicated that these mice displayed biliary hyperplasia and fibrosis (Fig 4) which appeared to be more severe than had previously been reported for FoxA1:FoxA2-null mice, HBVFoxA1^fl/fl^FoxA2^fl/fl^AlbCre(+) [47]. To more precisely address this issue, total liver RNA was analyzed by RT-qPCR to evaluate cytokine transcript levels, stellate cell activation and biliary epithelial proliferation (Fig 5). In all cases, FoxA2-null HBV transgenic mice (HBVFoxA2^fl/fl^AlbCre(+)) did not display any significant differences from control mice. In contrast, FoxA1:FoxA2-null mice (HBVFoxA1^fl/fl^FoxA2^fl/fl^AlbCre(+)) showed an intermediate phenotype between the control and the HBV transgenic mice expressing only reduced levels of FoxA3 (HBVFoxA1^fl/fl^FoxA2^fl/fl^FoxA3^+/-^AlbCre(+)). FoxA1:FoxA2-null mice (HBVFoxA1^fl/fl^FoxA2^fl/fl^AlbCre(+)) displayed a 2–4 fold increase in TNFα and 2OAS (a marker for type I interferon synthesis) mRNA compared to the controls (Fig 5A and 5B). HBV transgenic mice expressing only reduced levels of FoxA3 (HBVFoxA1^fl/fl^FoxA2^fl/fl^FoxA3^+/-^AlbCre(+)) displayed a 4–24 fold increase in the cytokine transcripts (Fig 5A and 5B). As these cytokines can post-transcriptionally reduce HBV replication intermediate levels [52–54], these observations may account for the differences in the reduction of HBV RNA and DNA in the HBVFoxA1^fl/fl^FoxA2^fl/fl^FoxA3^+/-^AlbCre(+) mice. IL6 mRNA levels displayed very modest changes in the various FoxA-deficient mice (Fig 5C). In contrast, TGFβ1, 2 and 3 levels were dramatically induced in the HBV transgenic mice expressing only reduced levels of FoxA3 (HBVFoxA1^fl/fl^FoxA2^fl/fl^FoxA3^+/-^AlbCre(+)) whereas the induction was much less dramatic in the FoxA1:FoxA2-null mice (HBVFoxA1^fl/fl^FoxA2^fl/fl^FoxA3^+/-^AlbCre(+)) (Fig 5D–5F). Similar quantitative differences were apparent for stellate cell activation as reflected in the induction of αSMA and Col1A1 mRNAs (Fig 5G and 5H) and biliary epithelial cell proliferation as seen by the induction of CK19 and 20 mRNAs (Fig 5I and 5J). These observations are all consistent with HBV transgenic mice expressing only reduced levels of FoxA3 (HBVFoxA1^fl/fl^FoxA2^fl/fl^FoxA3^+/-^AlbCre(+)) displaying a greater level of cytokine-mediate fibrosis and biliary hyperplasia than the FoxA1:FoxA2-null HBV transgenic mice (HBVFoxA1^fl/fl^FoxA2^fl/fl^AlbCre(+)) (Fig 5). Despite the FoxA-deficiency, mutant mice displayed the same level of RNA for key transcription factors (HNF1α, HNF1β, PPARα, HNF4α, FXRα and LRH1) know to govern HBV biosynthesis as were observed in the control mice (HBVFoxA1^fl^/^fl^FoxA2^fl^/^fl^FoxA3+/-AlbCre(-) relative to HBVFoxA1^fl^/^fl^FoxA2^fl^/^fl^FoxA3+/-AlbCre(+); fold difference = 0.8–1.3, p>0.2, n = 4 to 9). These observation suggest that transcription factor abundance within the liver does not explain the observed loss of HBV biosynthesis within the FoxA-deficient mice (HBVFoxA1^fl/fl^FoxA2^fl/fl^FoxA3^+/-^AlbCre(+))

### Effect of FoxA deficiency on viral transcription in HBV transgenic mice during postnatal development

**Fig 6:**
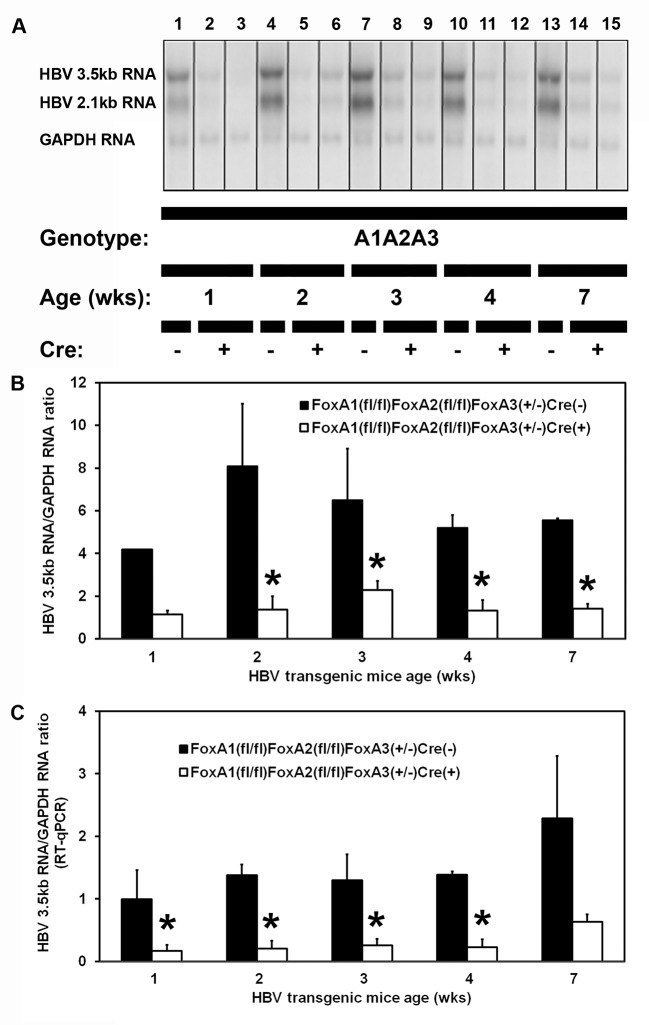
RNA (Northern) filter hybridization and RT-qPCR analysis of HBV transcripts in the livers of 1, 2, 3, 4 and 7 week old HBV transgenic mice. (A) RNA (Northern) filter hybridization analysis of representative mice of each genotype are shown. Noncontiguous lanes from multiple analysis are presented. The probes used were HBV*ayw* genomic DNA plus GAPDH cDNA. FoxA-expressing (HBVFoxA1^fl/fl^FoxA2^fl/fl^FoxA3^+/-^AlbCre(-)) and FoxA-deleted (HBVFoxA1^fl/fl^FoxA2^fl/fl^FoxA3^+/-^AlbCre(+)) HBV transgenic mice are indicated (Genotype A1A2A3). The glyceraldehyde 3-phosphate dehydrogenase (GAPDH) transcript was used as an internal control for the quantitation of the HBV 3.5kb RNA. (B) Quantitative analysis by RNA (Northern) filter hybridization of the HBV 3.5kb transcript in the HBV transgenic mice. The mean HBV 3.5kb transcript levels plus standard deviations are indicated. The levels of the transcripts which are statistically significantly different between Cre(-) and Cre(+) HBV transgenic mice by a Student’s t-test (p<0.05) are indicated with an asterisk (*). Average number of mice per group was 2.7±1.3 (Range: 2–5, except for the 1 week old control group where quantification of a single mouse is presented). (C) Quantitative analysis by RT-qPCR of the HBV 3.5kb transcript in the HBV transgenic mice. The mean HBV 3.5kb transcript levels plus standard deviations are indicated. The levels of the transcripts which are statistically significantly different between Cre(-) and Cre(+) HBV transgenic mice by a Student’s t-test (p<0.05) are indicated with an asterisk (*). Average number of mice per group was 3.5±1.7 (Range: 2–7).

As adult HBV transgenic mice expressing only reduced levels of FoxA3, HBVFoxA1^fl/fl^FoxA2^fl/fl^FoxA3^+/-^AlbCre(+), display fibrosis and biliary hyperplasia (Figs 4 and 5), it was of interest to determine when this phenotype became apparent during postnatal development and what effect it might have on HBV RNA synthesis (Fig 6). Initially, the level of HBV transcription throughout postnatal development was determined by RNA filter hybridization analysis and RT-qPCR analysis (Fig 6). As observed with adult mice (Fig 2), HBV transcription was reduced 3–7 fold in the HBV transgenic mice expressing only reduced levels of FoxA3 (HBVFoxA1^fl/fl^FoxA2^fl/fl^FoxA3^+/-^AlbCre(+)) compared with the controls throughout postnatal development.

### Effect of FoxA deficiency on cytokine production, stellate cell activation and biliary epithelial cell proliferation within the livers of HBV transgenic mice during postnatal development

**Fig 7:**
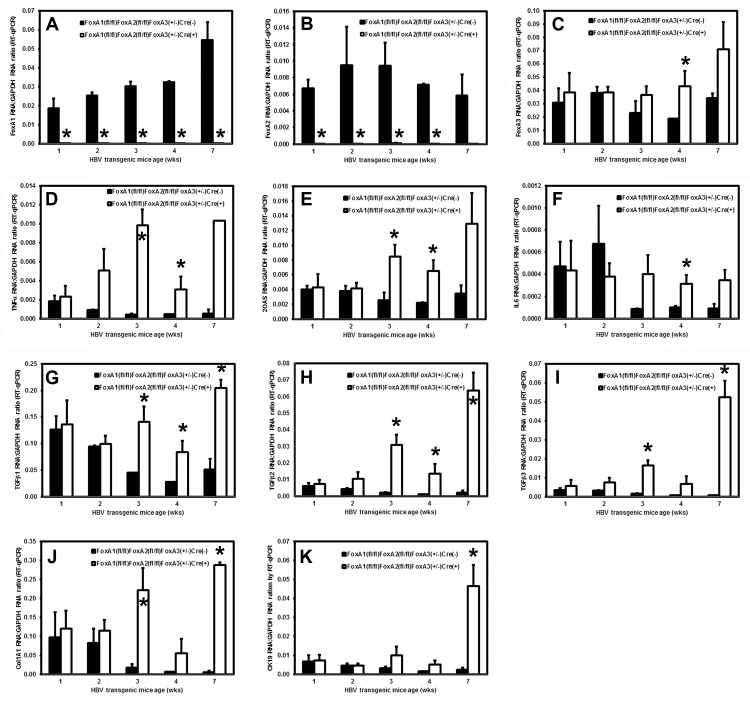
Effects of FoxA deletion on liver FoxA1, FoxA2, FoxA3, TNFα, 2OAS, IL6, TGFβ1, TGFβ2, TGFβ3, Col1A1 and CK19 transcript levels in 1, 2, 3, 4 and 7 week old HBV transgenic mice. Quantitative analysis of the (A) FoxA1, (B) FoxA2, (C) FoxA3, (D) TNFα, (E) 2OAS, (F) IL6, (G) TGFβ1, (H) TGFβ2, (I) TGFβ3, (J) Col1A1 and (K) CK19 transcripts by RT-qPCR in the HBV transgenic mice. The GAPDH transcript was used as an internal control for the quantitation of the FoxA1, FoxA2, FoxA3, TNFα, 2OAS, IL6, TGFβ1, TGFβ2, TGFβ3, Col1A1 and CK19 RNAs. The mean relative FoxA1, FoxA2, FoxA3, TNFα, 2OAS, IL6, TGFβ1, TGFβ2, TGFβ3, Col1A1 and CK19 transcript levels plus standard deviations derived from FoxA-expressing (HBVFoxA1^fl/fl^FoxA2^fl/fl^FoxA3^+/-^AlbCre(-)) and FoxA-deleted (HBVFoxA1^fl/fl^FoxA2^fl/fl^FoxA3^+/-^AlbCre(+)) HBV transgenic mice is shown. The levels of the transcripts which are statistically significantly different between Cre(-) and Cre(+) HBV transgenic mice by a Student’s t-test (p<0.05) are indicated with an asterisk (*). Average number of mice per group was 3.5±1.7 (Range: 2–7).

To address the timing of the postnatal alterations associated with FoxA deficiency, total liver RNA was analyzed by RT-qPCR to evaluate cytokine transcript levels, stellate cell activation and biliary epithelial proliferation at 1, 2, 3, 4 and 7 weeks of age in wildtype HBV transgenic mice, HBVFoxA1^fl/fl^FoxA2^fl/fl^FoxA3^+/-^AlbCre(-), and HBV transgenic mice expressing only reduced levels of FoxA3, HBVFoxA1^fl/fl^FoxA2^fl/fl^FoxA3^+/-^AlbCre(+) (Fig 7). The complete loss of FoxA1 and FoxA2 RNA was apparent in the livers of 1 week old HBV transgenic mice expressing only reduced levels of FoxA3 (HBVFoxA1^fl/fl^FoxA2^fl/fl^FoxA3^+/-^AlbCre(+)) (Fig 7A and 7B). By 4 weeks, FoxA3 RNA levels were higher in the FoxA-deficient mice (HBVFoxA1^fl/fl^FoxA2^fl/fl^FoxA3^+/-^AlbCre(+)) as compared with the wildtype mice (HBVFoxA1^fl/fl^FoxA2^fl/fl^FoxA3^+/-^AlbCre(-) (Fig 7C). For the markers of inflammation, biliary epithelial cells proliferation and fibrosis, no significant differences in mRNA levels were observed at 1 or 2 weeks of age between control and FoxA-deficient mice (HBVFoxA1^fl/fl^FoxA2^fl/fl^FoxA3^+/-^AlbCre(-) and HBVFoxA1^fl/fl^FoxA2^fl/fl^FoxA3^+/-^AlbCre(+), respectively) indicating that major differences in these processes were not apparent until the FoxA-deficient mice (HBVFoxA1^fl/fl^FoxA2^fl/fl^FoxA3^+/-^AlbCre(+)) were 3 weeks old. This suggests that the elevated levels of all these markers which are apparent during this neonatal stage of liver development are a normal part of the process of neonatal liver growth (Fig 7D–7K). In contrast, after 2 weeks of age the levels of cytokine RNAs (Fig 7D–7I), biliary epithelial cells proliferation markers (Fig 7K) and fibrosis associated genes (Fig 7J) increase in the FoxA-deficient mice (HBVFoxA1^fl/fl^FoxA2^fl/fl^FoxA3^+/-^AlbCre(+)) whereas their levels generally decrease as liver growth slows in the wildtype mice (HBVFoxA1^fl/fl^FoxA2^fl/fl^FoxA3^+/-^AlbCre(-)). As HBV transcription is already decreased in the 1 and 2 week old FoxA-deficient HBV transgenic mice (HBVFoxA1^fl/fl^FoxA2^fl/fl^FoxA3^+/-^AlbCre(+)) relative to the controls (HBVFoxA1^fl/fl^FoxA2^fl/fl^FoxA3^+/-^AlbCre(-)) (Fig 6), it appears that FoxA-deficiency alone is sufficient to reduce viral transcription.

### Effect of FoxA deletion on HBV DNA methylation in the livers of HBV transgenic mice

**Fig 8:**
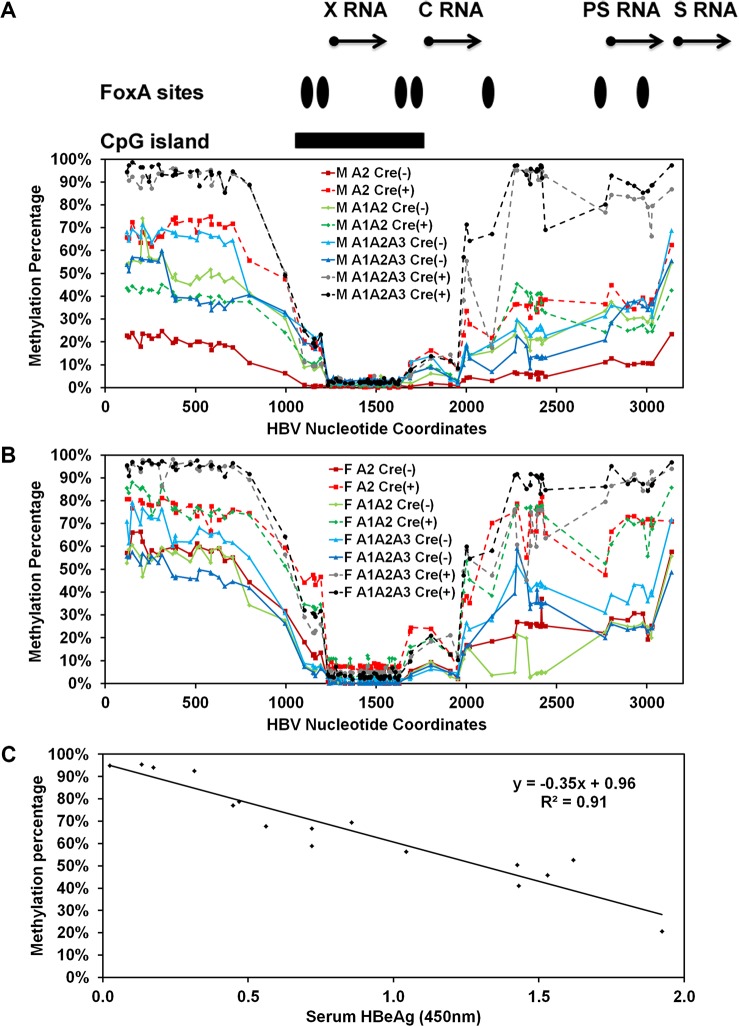
Effect of FoxA-deletion on HBV DNA methylation in adult mouse liver. The percentage of CpG DNA methylation at each position within the HBV genome from male (A) and female (B) FoxA-expressing (HBVFoxA2^fl/fl^AlbCre(-), HBVFoxA1^fl/fl^FoxA2^fl/fl^AlbCre(-) and HBVFoxA1^fl/fl^FoxA2^fl/fl^FoxA3^+/-^AlbCre(-)) and FoxA-deleted (HBVFoxA2^fl/fl^AlbCre(+), HBVFoxA1^fl/fl^FoxA2^fl/fl^AlbCre(+) and HBVFoxA1^fl/fl^FoxA2^fl/fl^FoxA3^+/-^AlbCre(+)) HBV transgenic mice is shown. The positions of the viral transcription initiation sites for the X-gene (X RNA), the precore/pregenomic transcripts (C RNA), the large surface antigen transcript (PS RNA) and the middle/major surface antigen transcript (S RNA) are shown. The locations of the FoxA binding sites and CpG island within the HBV genome are also indicated. (C) The average percent methylation of the CpG sites spanning nucleotide coordinate 1–706 is correlated with the level of serum HBeAg.

FoxA-deficiency in the HBV transgenic mice expressing only reduced levels of FoxA3, HBVFoxA1^fl/fl^FoxA2^fl/fl^FoxA3^+/-^AlbCre(+), is associated with a dramatic developmental reduction in HBV RNA and DNA synthesis (Figs 2, 3, 4 and 6). As FoxA is a pioneer transcription factor responsible for marking liver-specific genes for expression later in hepatocyte maturation and DNA methylation contributes to tissue specific gene expression, it was of interest to see if FoxA-deficiency was affecting HBV gene expression by altering the developmental pattern of viral DNA methylation. Consequently, bisulfite genomic sequencing of the HBV transgene DNA from wild-type and FoxA gene deleted HBV transgenic mice was performed to determine the role of FoxA in the regulation of viral DNA methylation (Fig 8). Regardless of FoxA status, the CpG island located between HBV nucleotide coordinates 1066–1773 (%GC = 54.9, Observed CpG/Expected CpG = 0.85, Length = 708bp) was hypomethylated (Fig 8A and 8B) [55]. This region encompasses the enhancer 1/X-gene promoter and enhancer 2/core gene promoter regions and is flanked by two FoxA binding sites [17]. The percentage of DNA methylation increases progressively from approximately nucleotide coordinate 2000–3182 which includes the large and major surface antigen promoter and three additional FoxA binding sites [40, 41]. The percentage of DNA methylation is at its highest and most consistent levels on all genomes from approximately nucleotide coordinate 1–706 (Fig 8A and 8B). Importantly, the average level of HBV DNA methylation between nucleotide coordinates 1–706 for all 16 mice examined in this analysis correlates (R^2^ = 0.91) strongly with the level of serum HBeAg (Fig 8C) suggesting that this region, at a minimum, plays an important role in governing the level of HBV 3.5kb precore RNA synthesis and hence HBeAg production. Given that this region of the viral genome has not previously been identified as playing a role in governing HBV RNA synthesis, it appears that this may represent a previously unknown *in vivo* mode of HBV transcriptional regulation. Furthermore, it is apparent that the inhibition of HBV transcription that is apparent in the HBV transgenic mice expressing only reduced levels of FoxA3, HBVFoxA1^fl/fl^FoxA2^fl/fl^FoxA3^+/-^AlbCre(+), correlates with the very high level of methylation (93–95%) observed in this region of the genome suggesting that DNA methylation resulting from FoxA-deficiency is associated with the observed loss of viral transcription and replication intermediates (Figs 2 and 3). Interestingly, the control, FoxA2-null and FoxA1:Fox2-null mice (HBVFoxA2^fl/fl^AlbCre(+) and HBVFoxA1^fl/fl^FoxA2^fl/fl^AlbCre(+), respectively) display a range of DNA methylation levels between 20–80% which is consistent with the variation in serum HBeAg (Fig 8C) and appears to be consistent with the number of hepatocytes expressing HBcAg by immunohistochemistry (Fig 4).

**Fig 9:**
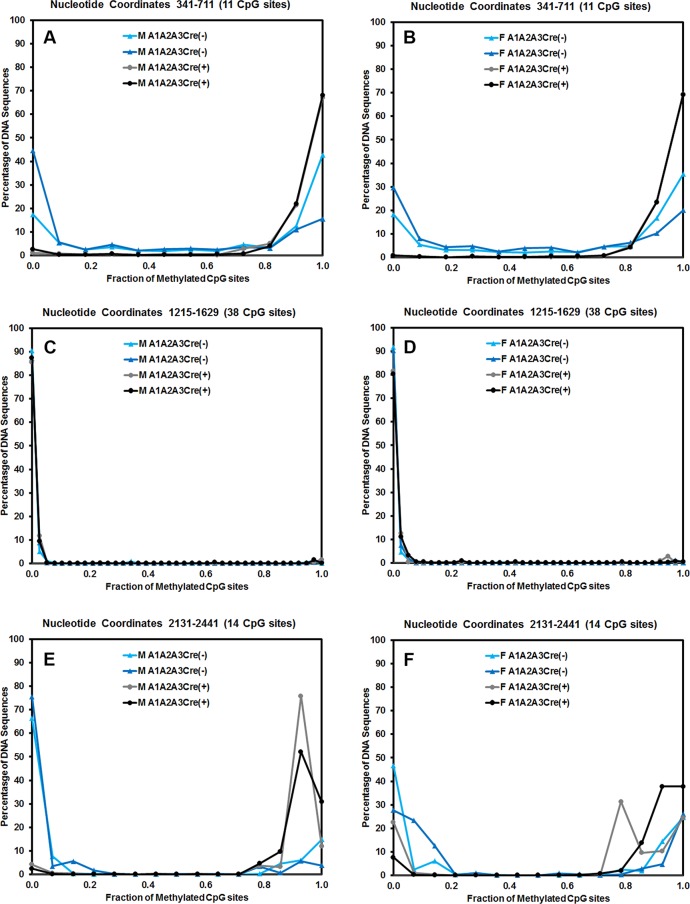
Effect of FoxA-deletion on HBV DNA methylation distribution in adult mouse liver. The CpG DNA methylation frequency distribution across the 11, 38 and 14 sites within HBV nucleotide coordinates 341–711, 1215–1629 and 2131–2441, respectively, from male (A, C and E) and female (B, D and F) FoxA-expressing (HBVFoxA1^fl/fl^FoxA2^fl/fl^FoxA3^+/-^AlbCre(-); M A1A2A3Cre(-) and F A1A2A3Cre(-)) and FoxA-deleted (HBVFoxA1^fl/fl^FoxA2^fl/fl^FoxA3^+/-^AlbCre(+); M A1A2A3Cre(+) and F A1A2A3Cre(+)) HBV transgenic mice is shown.

To address this issue, the distribution of DNA methylation sites within the three HBV DNA amplicons that contained the largest number of CpG sites were evaluated for the control, HBVFoxA1^fl/fl^FoxA2^fl/fl^FoxA3^+/-^AlbCre(-), and HBV transgenic mice expressing only reduced levels of FoxA3, HBVFoxA1^fl/fl^FoxA2^fl/fl^FoxA3^+/-^AlbCre(+) (Fig 9). The HBV DNA amplicon spanning nucleotide coordinates 341–711 contains 11 CpG sites (Fig 9A and 9B). The percentage of DNA sequences that were hypermethylated (fully methylated or unmethylated at only a single site) correlated with the overall level of methylation of this sequence at each position. This indicates that regardless of the origin of the HBV transgene DNA, it was either completely (or almost completely) methylated or completely (or almost completely) unmethylated (Fig 9A and 9B). Virtually none of the sequences displayed an intermediate level of DNA methylation. This is consistent with the suggestion that this region of the genome is unmethylated in the hepatocytes that are expressing the HBV transgene DNA and hence supporting viral biosynthesis but extensively methylated in the hepatocytes that are not expressing the HBV transgene DNA and hence are not supporting viral biosynthesis. These observations are consistent with previous immunohistochemical analysis of HBcAg in HBV transgenic mouse livers [21, 49, 50].

The HBV DNA amplicon spanning nucleotide coordinates 1215–1629 contains 38 CpG sites (Fig 9C and 9D). This HBV transgene DNA amplicon is located within the CpG island and as such is hypomethylated [56]. Methylation of any CpG sequence within this region was very rare as might be expected (Fig 8) so almost all sequences were unmethylated with only a very limited number of singly methylated CpG sequence.

The HBV DNA amplicon spanning nucleotide coordinates 2131–2441 contains 14 CpG sites (Fig 9E and 9F). As noted for the HBV DNA amplicon spanning nucleotide coordinates 341–711, the HBV transgene DNA was either hypo- or hypermethylated with up to 3 CpG sites being methylated or unmethylated, respectively, within any single amplicon sequence. This observation is again consistent with the suggestion that the HBV transgene DNA is either almost completely methylated or completely unmethylated within this region of the genome (Fig 9E and 9F). Partial methylation of the HBV transgene DNA is essentially absent suggesting that methylation of one CpG site enhances the probability that adjacent CpG sites will also be methylated.

### Effect of tissue specificity and liver development on methylation of HBV transgene DNA

**Fig 10:**
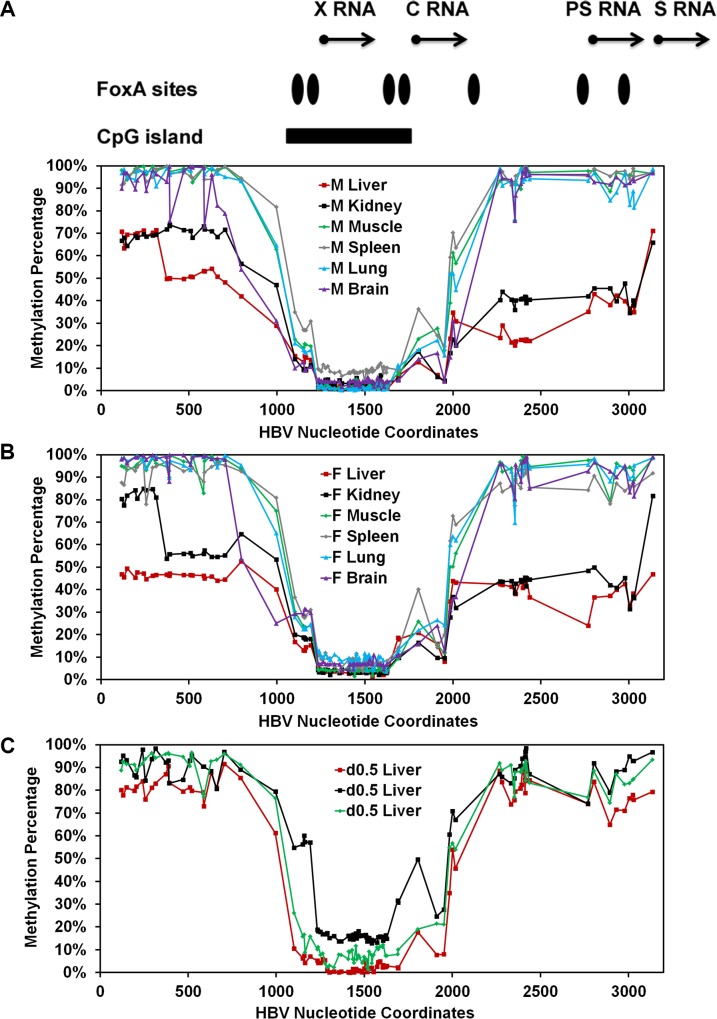
Tissue-specific and developmental regulation of HBV DNA methylation. (A) The percentage of CpG DNA methylation at each position within the HBV genome from a male (A) and female (B) wild-type HBV transgenic mouse liver, kidney, muscle, spleen, lung and brain is shown. The positions of the viral transcription initiation sites for the X-gene (X RNA), the precore/pregenomic transcripts (C RNA), the large surface antigen transcript (PS RNA) and the middle/major surface antigen transcript (S RNA) are shown. The locations of the FoxA binding sites and CpG island within the HBV genome are also indicated. (C) The percentage of CpG DNA methylation at each position within the HBV genome from three individual 0.5 day old neonatal wild-type HBV transgenic mouse livers is shown.

In the transgenic mouse model of chronic HBV infection, HBV transcription occurs in a limited number of tissues including the liver and kidney [21, 57]. In contrast, HBV transcription is essentially absent from other tissues including the muscle, spleen, lung and brain [21, 57]. To evaluate the role of methylation in the tissue specific expression of the HBV transgene, the level of HBV transgene DNA methylation was evaluated in these tissues (Fig 10). Regardless of the tissue being examined, the CpG island located between HBV nucleotide coordinates 1066–1773 was hypomethylated (Fig 10A and 10B). Similar to the findings in the livers of the HBV transgenic mice expressing only reduced levels of FoxA3, HBVFoxA1^fl/fl^FoxA2^fl/fl^FoxA3^+/-^AlbCre(+) (Fig 8), where viral transcription was limited, the regions of the HBV genome spanning nucleotide 2000–3182 and 1–706 were hypermethylated in muscle, spleen, lung and brain (Fig 10A and 10B) consistent with a role for DNA methylation in the inhibition of viral transcription in these tissues. Of note, these same regions of the viral genome were only partially methylated in liver and kidney, the tissues that supports relatively abundant HBV transcription [21, 57].

**Fig 11:**
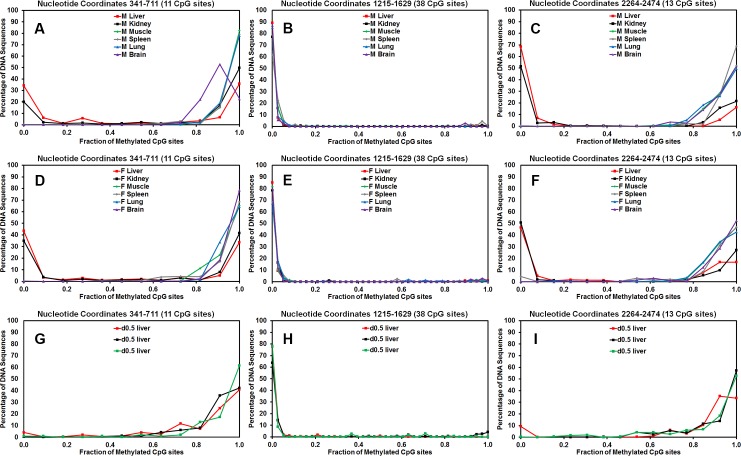
Tissue-specific and developmental regulation of HBV DNA methylation distribution. The CpG DNA methylation frequency distribution across the 11, 38 and 13 sites within HBV nucleotide coordinates 341–711, 1215–1629 and 2264–2474, respectively, from male (A-C) and female (D-F) wild-type HBV transgenic mouse liver, kidney, muscle, spleen, lung and brain DNA is shown. The CpG DNA methylation frequency distribution across the 11, 38 and 13 sites within HBV nucleotide coordinates 341–711, 1215–1629 and 2264–2474, respectively, from three independent 0.5 day old neonatal (G-I) wild-type HBV transgenic mouse liver DNA is shown.

As HBV biosynthesis is essentially absent in wildtype neonates [19], it was of interest to investigate the methylation status of HBV genomic DNA from the livers of new born mice (Fig 10C). The methylation profile of the HBV genomic DNA from these wildtype neonates was essentially the same as the livers of HBV transgenic mice expressing only reduced levels of FoxA3, HBVFoxA1^fl/fl^FoxA2^fl/fl^FoxA3^+/-^AlbCre(+) (Fig 8) and the transgene DNA from muscle, spleen, lung and brain (Fig 10A and 10B). Furthermore, the HBV DNA amplicon spanning nucleotide coordinates 341–711, 1215–1629 and 2264–2474 (Fig 11) demonstrate that the HBV transgene DNA is either hypo- or hypermethylated within any singe amplicon sequence. This observation is again consistent with the suggestion that the HBV transgene DNA is either almost completely methylated or completely unmethylated within these regions of the genome regardless of the origin of the HBV transgene DNA (Fig 11). In addition, the level of methylation within the region spanning nucleotide coordinates 341–711 correlates with the level of HBV transcription in the various tissues and at distinct postnatal periods of liver development. This suggests that FoxA may govern the developmental expression of the HBV genome by modulating the level of viral DNA methylation throughout hepatocyte maturation.

## Discussion

The epigenetic modification of eukaryotic genomic DNA by methylation of cytosine residues in the context of CpG sites has been characterized in some detail throughout development [58, 59]. During gametogenesis and in the pre-implantation embryo, DNA is demethylated and subsequently remethylated as the process of early development proceeds through the initial stages of cellular proliferation and germ cell layer differentiation [58, 59]. Appropriate methylation of genomic DNA during development is essential for viability with the loss of DNA methyltransferase (DNMT) activity in mice being associated with an embryonic or postnatal lethal phenotype [60, 61]. Furthermore, it is apparent that when cells differentiate into tissue-specific cell types, the patterns of RNA expression observed in these cells negatively correlates to a significant degree with the methylation status of the DNA sequence elements in proximity to the associated gene [35]. Hypomethylation of regulatory DNA sequence elements, including enhancer, promoter and intragenic sequences, are generally associated with expressed genes whereas hypermethylation of regulatory DNA sequence elements is primarily associated with non-expressed genes [35, 62]. However, detailed mechanistic insights into the developmental processes leading to these general patterns of gene expression and DNA methylation is currently quite limited [36, 37, 62].

Pioneer transcription factors are critical determinants of tissue specific gene expression [26]. Pioneer transcription factors bind to the regulatory regions of genes and mark them for expression at a later stage of development [29]. Pioneer factors can bind to chromatin in the context of nucleosomes, leading to a more relaxed conformation which permits gene expression to occur once additional transcription factors bind to the enhancer and promoter regions of these genes at later developmental stages [25]. However, it is not clear why the absence of pioneer factor binding to non-target genes leads to the complete failure of gene expression at subsequence stages of cellular differentiation. It does suggest that the genes that are not marked by pioneer factors in a specific cell type must, somehow, be identified in a manner which prevents their subsequent activation regardless of the expression of additional transcription factors at later stages in development and/or differentiation. Processing these genes into heterochromatin would be one route that would ensure the silencing of these genes and this might be aided by the hypermethylation of the regions of the chromosomes containing the relevant genes in the appropriate cells of any particular tissue [63]. However, cell-type specific deletion of pioneer factors which are essential for the correct gene expression patterns to mediate cellular differentiation is not possible because of the essential nature of their target genes for functional tissue development [26]. Consequently there is only limited understanding of the mechanism(s) of pioneer factor function and the consequences of their loss in the tissues where they play a major role in tissue development. Therefore a non-essential target gene which is highly dependent on pioneer factor expression for its developmental expression may be informative if conditions exist where the target gene but not the essential cellular genes are completely dependent on a specific regulated pattern of pioneer transcription factor expression.

The HBV genome contains seven binding sites for the FoxA pioneer transcription factor and all four of the viral promoters are regulated by FoxA in cell culture [39–41]. Furthermore, FoxA modulates HBV transcription and replication in non-hepatoma cells complemented with nuclear receptors [16, 42]. Therefore it was of interest to determine the role of this pioneer factor in the regulation of HBV transcription and replication *in vivo* using the HBV transgenic mouse model of chronic viral infection in the presence and absence of various degrees of FoxA-deficiency. Consequently, liver-specific FoxA2-null, liver-specific FoxA1-null:FoxA2-null and liver-specific FoxA1:FoxA2-null:FoxA3-heterozygous HBV transgenic mice (HBVFoxA2^fl/fl^AlbCre(+), HBVFoxA1^fl/fl^FoxA2^fl/fl^AlbCre(+) and HBVFoxA1^fl/fl^FoxA2^fl/fl^FoxA3^+/-^AlbCre(+), respectively), were generated and characterized. The most dramatic phenotype was associated with the liver-specific FoxA1:FoxA2-null:FoxA3-heterozygous HBV transgenic mice, HBVFoxA1^fl/fl^FoxA2^fl/fl^FoxA3^+/-^AlbCre(+), which express only reduced levels of FoxA3 (Fig 1A–1C). These mice have enlarged livers (Fig 1E) but very low levels of serum HBeAg (Fig 1F). They have reduced levels of viral RNA and DNA in their livers (Figs 2 and 3), which explains the low level of serum HBeAg and indicates that FoxA is essential for the *in vivo* expression of the HBV genomic DNA. Immunohistochemical analysis of the livers of these mice revealed dramatically reduced levels of HBcAg (Fig 4A and 4D), which accounts for the complete loss of viral DNA replication intermediates (Fig 3). Furthermore, these livers displayed biliary hyperplasia and fibrosis to a greater extent than previously reported in the liver-specific FoxA1:FoxA2-null mice (HBVFoxA1^fl/fl^FoxA2^fl/fl^AlbCre(+)) (Figs 4B, 4C, 4E, 4F and 5) [47]. The adult HBV transgenic mice which express only reduced levels of FoxA3 (HBVFoxA1^fl/fl^FoxA2^fl/fl^FoxA3^+/-^AlbCre(+)) also displayed elevated levels of transcripts for a number of cytokines including TNFα, 2OAS as an indicator of type I interferon expression, IL6 and TGFβ (Fig 5A–5F) which may have influenced the levels of HBV replication intermediates by post-transcriptional mechanisms [51]. To address this issue, viral transcription was assessed in the livers of neonatal HBV transgenic mice between the ages of one to four weeks (Fig 6). Differences in cytokine transcript levels were not apparent until mice were 3 weeks of age (Fig 7) whereas FoxA-deficiency (HBVFoxA1^fl/fl^FoxA2^fl/fl^FoxA3^+/-^AlbCre(+)) was associated with reduced HBV transcription at all developmental stages examined (Fig 6). These observations indicate that FoxA is essential for HBV transcription throughout postnatal liver development.

**Fig 12:**
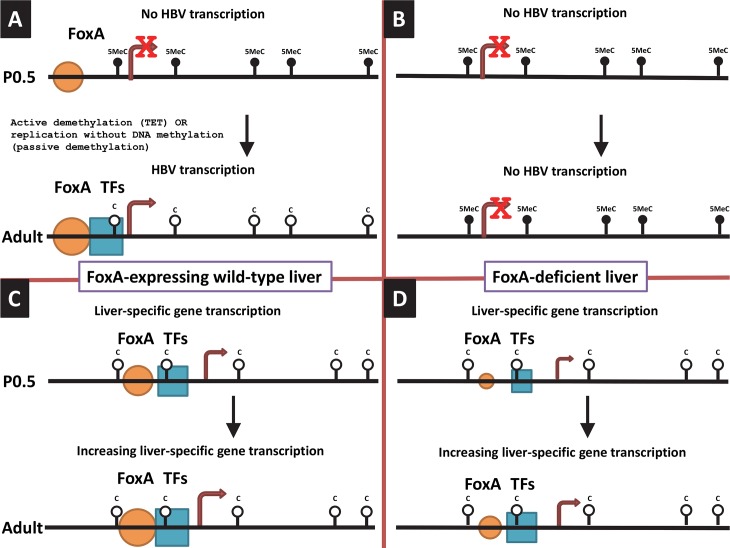
Model for the tissue-specific and developmental regulation of HBV DNA methylation and transcription by the pioneer transcription factor, FoxA. (A) In the neonatal (P0.5) wild-type HBV transgenic mice, the HBV transgene DNA is extensively methylated but FoxA is expressed and marks the HBV genome for later developmental expression upon recruitment of additional transcription factors to the viral promoters. In the adult wild-type HBV transgenic mice, the HBV transgene DNA is unmethylated, FoxA plus additional transcription factors are recruited to the viral promoters and HBV RNA and DNA synthesis is observed. (B) In the neonatal (P0.5) FoxA-deficient HBV transgenic mice, the HBV transgene DNA is extensively methylated while limiting levels of FoxA fail to mark the HBV genome for later developmental DNA demethylation (due to the failure to recruit TET for active DNA demethylation and/or inhibition of DNA methylation during replication), and hence subsequent recruitment of additional transcription factors to the viral promoters with concomitant viral gene expression. In the adult FoxA-deficient HBV transgenic mice, the HBV genome remains extensively methylated because the limiting abundance of FoxA throughout development fails to mark the viral transgene DNA for demethylation which is essential for HBV RNA and DNA synthesis. (C) In the neonatal (P0.5) wild-type HBV transgenic mice, the essential liver-specific genes which are dependent on FoxA for their expression at this stage of development have presumably been marked by this pioneer transcription factor leading to the recruitment of additional transcription factors necessary for the demethylation and subsequent expression of these genes. Increasing levels of liver-specific transcription factors associated with liver maturation may be associated with increased levels of gene expression in the adult mice. (D) In contrast to the effect of limiting FoxA abundance on HBV DNA methylation and transcription, limiting postnatal FoxA abundance in the hepatocytes of these mice must be sufficient to support gene expression levels consistent with host viability presumably by marking these genes for DNA demethylation and transcription at the neonatal stage of development. The size of the transcription factors (FoxA and TFs) reflects the number of hepatocytes expressing HBV or essential liver-specific genes and/or the overall level of gene expression. The size of the arrow reflects the level of gene transcription. 5MeC, 5-methylcytosine; C, cytosine.

Methylation analysis of HBV transgene DNA from adult mice demonstrated that FoxA-deficiency (HBVFoxA1^fl/fl^FoxA2^fl/fl^FoxA3^+/-^AlbCre(+)) increased viral epigenetic modification at CpG sites within the region spanning nucleotide coordinates 1–706 (and to a less extent 2000–3182) such that it was approaching 100% (Fig 8). The level of CpG site methylation within the region from 1–706 correlated with the level of serum HBeAg (Fig 8C) and was consistent with the immunohistochemical distribution of HBcAg within the liver lobules of these mice (Fig 4A and 4D). These observations suggested that the HBV transgene DNA within the hepatocytes expressing HBV transcripts and replicating virus were essentially unmethylated whereas the hepatocytes failing to support HBV transcription and replication possessed HBV transgene DNA which was hypermethylated. This appears to be the case in all mice as the frequency distribution of DNA methylation was essentially all or none within the HBV DNA amplicons spanning nucleotide coordinates 341-711and 2131–2441 (Fig 9). Similar analysis of neonatal livers isolated from 0.5 day old wild type HBV transgenic mice and additional adult tissues (Fig 10) also indicated that only adult kidney and liver tissues which were capable of supporting viral transcription contained a fraction of cells which were hypomethylated within the HBV DNA amplicons spanning nucleotide coordinates 341-711and 2264–2474 (Fig 11). Together, these data indicate that FoxA is required to mark HBV genomic DNA for transcription by protecting it from DNA methylation or mediating its demethylation during the postnatal stages of liver development (Fig 12). Indeed, the observation that neonatal HBV transgene DNA from wildtype mice is essentially 100% methylated in the HBV DNA amplicons spanning nucleotide coordinates 341-711and 2264–2474 (Fig 11) but completely unmethylated in a fraction of hepatocytes in adult mice (Fig 11) indicates that the HBV transgene DNA must lose it 5-methylcytosine residues as a result of neonatal hepatocyte proliferation or maturation. This implies that the developmental binding of FoxA to HBV recognition sequences must either prevent the recruitment of DNMTs to the viral transgene DNA or actively recruit ten eleven translocation (TET) methylcytosine dioxygenases [64]. In the former case, DNA methylation is lost by subsequent genomic DNA replication associated with hepatocyte proliferation and in the latter demethylation is an active process leading to the removal of 5-methylcytosine by its oxidation to 5-hydroxymethylcytosine, 5-formylcytosine and 5-carboxylmethylcytosine followed by base-excision repair [65]. Alternatively, active FoxA-dependent DNA demethylation might involve deamination of 5-methylcytosine to thymine by activation-induced deaminase (AID) or apolipoprotein B mRNA-editing enzyme, catalytic polypeptides (APOBEC) followed by base-excision repair (BER) [66]. The kinetics of the loss of 5-methylcytosine during postnatal liver development should indicate if the process is active or passive and what, if any, role the TET enzymes or single- and double-strand DNA break repair pathways play in this process. If TET or BER are involved in HBV DNA demethylation, TET or BER inhibitors could potentially be considered as antiviral therapies for both neonatal infections and the treatment of chronic HBV infections in adults although secondary effects of such approaches would need careful evaluation.

This study demonstrates that FoxA is essential for postnatal HBV transcription and replication whereas under the specific conditions of limited FoxA3 expression seen in this transgenic mouse model (HBVFoxA1^fl/fl^FoxA2^fl/fl^FoxA3^+/-^AlbCre(+)), cellular gene expression remains sufficient to support viability and essentially normal hepatocyte function as reflected by serum chemistry with the exception of circulating cholesterol which is decreased about two-fold in these mice. Regardless, this indicates that limited postnatal FoxA3 expression is sufficient to support liver function and normal growth in the HBV transgenic mice. In contrast, the complete absence of FoxA expression in the liver is incompatible with viability. This indicates that limiting abundance of FoxA3 (HBVFoxA1^fl/fl^FoxA2^fl/fl^FoxA3^+/-^AlbCre(+)) can supply the pioneer function necessary for liver-specific gene expression necessary for essentially normal hepatocyte differentiation whereas it is insufficient for HBV transcription and replication due to its failure to protect the HBV transgene DNA from comprehensive region specific CpG site methylation. These observations imply that the pioneer factor function of FoxA are distinct for different genes and likely depend on FoxA concentrations through liver development in combination with the number and affinity of the FoxA recognition sequences associated with the genes it regulates through liver specification and differentiation (Fig 12).

Physiologically normal levels of FoxA expression during development result in the expression of HBV transcription and replication within a day of birth [19]. At birth, HBV transgene DNA is fully methylated (Figs 10 and 11). This suggests that immediately after birth, FoxA directly or indirectly inhibits methylation of the HBV genome as the hepatocytes proliferate and differentiate with liver growth. This may be achieved by FoxA directly or indirectly recruiting TET or BER proteins leading to the active demethylation of the HBV transgene DNA [64, 66]. Alternatively, FoxA may directly or indirectly prevent the recruitment of DNMTs to the HBV transgene DNA and passive demethylation may occur through multiple rounds of cellular DNA replication and cell division (Fig 12A). In the presence of a limiting abundance of FoxA3 (HBVFoxA1^fl/fl^FoxA2^fl/fl^FoxA3^+/-^AlbCre(+)) in the hepatocytes, this fails to occur and DNA methylation is preserved through cellular DNA replication and methylation of the hemi-methylated HBV transgene DNA by the maintenance DNA methyltransferase, DNMT1 (Fig 12B). Hypermethylated HBV DNA is transcriptionally inactive in natural infection, HBV transgenic mice and in cell culture [67–69]. Under normal cellular conditions, liver-specific genes are marked by FoxA during hepatocyte specification and differentiation and expressed in the liver at various developmental stages depending on the specific gene being considered (Fig 12C) [26, 29]. In contrast to the situation with the HBV transgene DNA, limiting abundances of FoxA3 (HBVFoxA1^fl/fl^FoxA2^fl/fl^FoxA3^+/-^AlbCre(+)) are sufficient to maintain the pioneer function of this transcription factor and establish levels of liver-specific gene expression which are at least sufficient to support essentially normal hepatocyte function for the lifetime of the mouse (Fig 12D). These observation indicate that probably both the level of FoxA expression and the affinity of pioneer factor binding to its cognate recognition sequence within the regulatory region of the enhancers and promoters of the liver-specific genes it regulates determines whether or not FoxA can mark a specific gene for expression later in the developmental program. Also as a consequence of differences in affinity of FoxA for its cognate recognition sequence and the increased expression of FoxA throughout liver development [20], it is likely that different FoxA target genes are marked for subsequent expression at different stages of hepatocyte maturation. Indeed consistent with this suggestion, examples of hepatocyte-expressed FoxA-regulated genes including serum albumin (Alb), apolipoprotein AI (apoAI) and cytosolic phosphoenolpyruvate carboxykinase (PEPCK) are demethylated at distinct stages of liver maturation [25, 36, 70–74]. Furthermore, HBV may be particularly sensitive to FoxA-deficiency due to the high number of sites within the viral genome. Ultimately, it appears that limiting amounts of FoxA3 in HBVFoxA1^fl/fl^FoxA2^fl/fl^FoxA3^+/-^AlbCre(+) mice leads to its failure to mark the HBV transgene DNA resulting in its hypermethylation and loss of transcriptional competence. This failure to appropriately developmentally mark the hypermethylated HBV transgene DNA with the FoxA pioneer transcription factor may lead to the HBV transgene DNA being incorporated into a repressive chromatin environment such as heterochromatin. In such a chromatin environment, the liver-enriched transcription factors present within the hepatocytes would not be able to access the regulatory elements within the viral genome resulting in the loss of HBV biosynthesis as observed in the HBVFoxA1^fl^/^fl^FoxA2^fl^/^fl^FoxA3+/-AlbCre(+) mice.

Interestingly, both the FoxA2-null mice and the FoxA-deficient HBV transgenic mice (HBVFoxA1^fl/fl^FoxA2^fl/fl^AlbCre(+) and HBVFoxA1^fl/fl^FoxA2^fl/fl^FoxA3^+/-^AlbCre(+), respectively) are sensitive to bile acid toxicity [47, 75]. This is consistent with the observation that reduced levels of FoxA2 are associated with reduced levels of certain bile acid transporters and bile acid modifying activities [75]. As seen for HBcAg, HBV RNA and DNA, viral expression is observed adjacent to the central vein in zone 3 of the hepatic lobule (Fig 4) [21]. This suggests the possibility that there may be a gradient of FoxA activity within the developing mouse hepatic lobule restricting viral biosynthesis to this region of the liver by methylating and silencing the viral transgene DNA in the hepatocytes distal to the central vein. FoxA-deficiency may drastically reduce the zone where FoxA activity is sufficient to prevent the hypermethylation of the HBV transgene DNA (Figs 4, 8 and 9). A similar situation might also be occurring with the expression of FoxA target genes encoding bile acid modifying and transporter proteins but probably to a more modest extent than seen for HBV expression [75]. In the wild type mice there is sufficient FoxA across the liver lobule to support expression of the bile acid modifiers and transporters at a level which can prevent bile acid toxicity. In contrast the zone of hepatocytes may become more restricted when FoxA is limiting, resulting in overt toxicity when exposed to high concentrations of bile acids due to the restricted number of hepatocytes able to correctly transport these metabolites [75], presumably due to the increased methylation of the FoxA target genes encoding bile acid modifying and transporter genes. Similar, mechanisms involving liver-specific pioneer factors may be responsible for the zonal expression of a number of other liver-specific gene products.

Of major clinical importance, most HBV infections worldwide occur from mother to child at birth due to mixing of maternal and fetal blood [76, 77]. In the HBV transgenic mouse model of chronic viral infection, viral biosynthesis does not occur in the liver until after birth and increases throughout postnatal maturation due to increased HBV transcription which parallels the increase in liver-enriched transcription factor abundance associated with hepatocyte terminal differentiation [19–21]. As the situation is likely to be similar in man, the transient therapeutic reduction in the level of FoxA in neonates infected with HBV may lead to the irreversible methylation and transcriptional inactivation of the covalently closed circular HBV DNA (CCC DNA) generated from the initial mother to child infection at birth. Given the viability and essentially normal blood chemistry observed in the adult FoxA-deficient HBV transgenic mice (HBVFoxA1^fl/fl^FoxA2^fl/fl^FoxA3^+/-^AlbCre(+)), the transient reduction in the level of FoxA in neonates is unlikely to have any long term health consequences while eliminating viral biosynthesis. A similar approach may also be beneficial in the treatment of adult HBV chronic carries. Additionally, a more detailed understanding of the process by which FoxA deficiency eliminates HBV biosynthesis may lead to the identification of additional therapeutic targets, such as the DNMT, TET and BER enzymes, which when modulated appropriately may lead to the selective methylation of HBV CCC DNA and its transcriptional silencing. If successful, this approach could lead to the eradication of the viral replication intermediate, HBV CCC DNA, which is resistant to all current therapies.

A potential caveat regarding these observations arises from the differences in the transcriptional templates utilized in the HBV transgenic mouse model and natural infection in man. In the former, the template is a terminal-redundant chromosomally integrated copy of the viral genome whereas in natural infection it is HBV CCC DNA. Whether the nature of the transcriptional template or the developmental timing of natural infection affects the methylation status of the viral DNA under conditions of FoxA deficiency requires additional investigation. Irrespective of these caveats, these studies clearly demonstrate that FoxA mediates the developmental demethylation of the HBV genomic DNA leading to viral transcription and replication in the HBV transgenic mouse model of chronic HBV infection. These effects of FoxA on HBV transcription are independent of the expression of cytokines responsible for biliary epithelial cell proliferation or stellate cell activation during liver development.

## Materials and methods

### Transgenic mice

The production and characterization of the HBV transgenic mouse lineage 1.3.32 has been described (5). These HBV transgenic mice contain a single copy of the terminally redundant, 1.3-genome length copy of the HBVayw genome integrated into the mouse chromosomal DNA. High levels of HBV replication occur in the livers of these mice. The mice used in the breeding experiments were homozygous for the HBV transgene and were maintained on the SV129 genetic background (9).

The production and characterization of the floxed FoxA1 (*FoxA1*^fl/fl^), floxed FoxA2 (*FoxA2*^fl/fl^), FoxA3-null and albumin Cre (lineage B6.Cg-Tg(Alb-cre)21Mgn/J, Jackson Laboratory) transgene (AlbCre) mice has been described [47, 75, 78–82]. The FoxA1^fl/fl^Cre, FoxA2^fl/fl^Cre and FoxA3-null mice do not express FoxA1, FoxA2 and FoxA3 (also called hepatocyte nuclear factor 3α, 3β and 3γ or HNF3α, HNF3β and HNF3γ), respectively, in the liver (and extrahepatic tissues in the case of the FoxA3-null mice) after Cre-mediated excision of the FoxA1 and FoxA2 gene sequences, respectively, located between the loxP sites. The mice used in the breeding experiments were homozygous for their respective alleles and maintained on either the C57BL6;129J (FoxA1^fl/fl^ and FoxA2^fl/fl^) or SV129 (FoxA3-null and AlbCre) genetic backgrounds [19, 46, 47, 81, 83].

HBV transgenic mice (lineage 1.3.32) were bred with mice carrying the floxed FoxA1 (*FoxA1*^fl/fl^), floxed FoxA2 (*FoxA2*^fl/fl^), FoxA3-null (*FoxA3*^-/-^) alleles and the albumin Cre (lineage B6.Cg-Tg(Alb-cre)21Mgn/J, Jackson Laboratory) transgene (AlbCre) to generate HBVFoxA2^fl/fl^AlbCre, HBVFoxA1^fl/fl^FoxA2^fl/fl^AlbCre and HBVFoxA1^fl/fl^FoxA2^fl/fl^FoxA3^+/-^AlbCre transgenic mice. Littermate HBVFoxA2^fl/fl^, HBVFoxA1^fl/fl^FoxA2^fl/fl^ and HBVFoxA1^fl/fl^FoxA2^fl/fl^FoxA3^+/-^ transgenic mice without the AlbCre transgene were used as controls.

Mice were screened for the HBV transgene, the FoxA1^fl/fl^, FoxA2^fl/fl^, FoxA3-null alleles and the AlbCre transgene by polymerase chain reaction (PCR) analysis of tail DNA. Tail DNA was prepared by incubating 1 cm of tail in 500 μl of 100 mM Tris hydrochloride (pH 8.0), 200 mM NaCl, 5 mM EDTA, 0.2% (wt/vol) SDS containing 100 μg/ml Proteinase K for 16 to 20 hours at 55°C. Samples were centrifuged at 14,000 rpm in an Eppendorf 5417C microcentrifuge for 10 minutes and the supernatant was precipitated with 500 μl of isopropanol. DNA was pelleted by centrifugation at 14,000 rpm in an Eppendorf 5417C microcentrifuge for 10 minutes and subsequently dissolved in 100 μl of 5 mM Tris hydrochloride (pH 8.0), 1 mM EDTA. The HBV transgene was identified by PCR analysis using the oligonucleotides, 5’-TCGATACCTGAACCTTTACCCCGTTGCCCG-3’ (oligo XpHNF4-1, HBV coordinates 1133 to 1159) and 5’-TCGAATTGCTGAGAGTCCAAGAGTCCTCTT-3’ (oligo CpHNF4-2, HBV coordinates 1683 to 1658), and 1 μl of tail DNA. A PCR product of 551 bp indicated the presence of the HBV transgene. The FoxA1 wild type and floxed alleles were identified by PCR analysis using the oligonucleotides, 5’-CTGTGGATTATGTTCCTGATC-3’ (oligo FoxA1F) and 5’-GTGTCAGGATG CCTATCTGGT-3’ (oligo FoxA1R), and 1 μl of tail DNA. A PCR product of 290 bp indicated the wild type FoxA1 allele whereas a PCR product of 480 bp indicated the floxed FoxA1 allele. The FoxA2 wild type and floxed alleles were identified by PCR analysis using the oligonucleotides, 5’-CCCCTGAGTTGGCGGTGGT-3’ (oligo FoxA2F) and 5’-TTGCTCACGGAAGAGTAGCC-3’ (oligo FoxA2R), and 1 μl of tail DNA. A PCR product of 290 bp indicated the wild type FoxA2 allele whereas a PCR product of 450 bp indicated the floxed FoxA2 allele. The FoxA3 wild type and null alleles were identified by PCR analysis using the oligonucleotides, 5’-GGCAGTGCTTCCGGGTATGTA-3’ (oligo FoxA3F), 5’-GGGAAGAGGTCCATGATCCAT-3’ (oligo FoxA3R), and 5’-CAAAGCGCCATTCGCCATTCA-3’ (oligo FoxA3LacZ-3), and 1 μl of tail DNA. A PCR product of 196 bp indicated the wild type FoxA3 allele whereas a PCR product of 290 bp indicated the null FoxA3 allele. The AlbCre transgene was identified by PCR analysis using the oligonucleotides, 5’-CCAGCTAAACATGCTTCATCGTCG-3’ (oligo CRE-1) and 5’-ATTCTCCCACCGTCAGTACGTGAG-3’ (oligo CRE-2), and 1 μl of tail DNA. A PCR product of 300 base pairs indicated the presence of the Cre transgene. The samples were subjected to 42 amplification cycles involving denaturation at 94°C for 1 minute, annealing at 55°C for 1 minute, and extension from the primers at 72°C for 2 minutes. The 20 μl reaction conditions used were as described by the manufacturer (Gene Choice) and contained 2 units of Taq DNA polymerase.

HBV transgenic mice were fed normal rodent chow and water was available *ad libitum*. Mice were sacrificed and liver tissue was frozen in liquid nitrogen and stored at –70°C prior to DNA and RNA extraction.

### Ethics statement

All animal experiments were Institutional Animal Care and Use Committee (IACUC) approved and performed according to institutional guidelines with University of Illinois at Chicago Institutional Biosafety and Animal Care Committee approval (ACC Number: 14–025). All animal procedures were performed in the College of Medicine Research Building at the UIC and adhere to the policies of the NIH Office of Laboratory Animal Welfare (OLAW), the standards of the Animal Welfare Act, the Public Health Service Policy, and the Guide for the Care and Use of Laboratory Animals.

### HBV DNA and RNA analysis

Total DNA and RNA were isolated from liver of HBV transgenic mice as described (8,46). Protein-free DNA was isolated in an identical manner to the total DNA except the proteinase K digestion was omitted [49]. DNA (Southern) filter hybridization analyses were performed using 20 μg of *Hin*dIII digested DNA (46). Filters were probed with ^32^P-labeled HBV*ayw* genomic DNA (10) to detect HBV sequences. RNA (Northern) filter hybridization analyses were performed using 10 μg of total cellular RNA as described (46). Filters were probed with ^32^P-labeled HBV*ayw* genomic DNA to detect HBV sequences and mouse glyceraldehyde 3-phosphate dehydrogenase (GAPDH) cDNA to detect the GAPDH transcript used as an internal control (45). Filter hybridization analyses were quantified by phosphorimaging using a Packard Cyclone Storage Phosphor System.

Reverse transcription-quantitative polymerase chain reaction (RT-qPCR) was used to measure the relative levels of FoxA1, FoxA2, FoxA3, FoxO1, tumor necrosis factor α (TNFα), 2’,5’-oligoadenylate synthase (2OAS), interleukin 6 (IL6), transforming growth factor β1 (TGFβ1), TGFβ2, TGFβ3, alpha smooth muscle actin (αSMA), collagen 1A1 (Col1A1), cytokeratin 19 (CK19), CK20 and HBV 3.5kb transcript levels in mouse liver RNA. After DNase I treatment, 1 μg of RNA was used for cDNA synthesis using the TaqMan reverse transcription reagents (Applied Biosystems, Foster City, CA), followed by real-time PCR quantification using SYBR Green and an Applied Biosystems 7300 real-time thermocycler (Applied Biosystems). Thermal cycling consisted of an initial denaturation step for 10 min at 95°C followed by 40 cycles of denaturation (15 sec at 95°C) and annealing/extension (1 min at 60°C). The relative FoxA1, FoxA2, FoxA3, FoxO1, TNFα, 2OAS, IL6, TGFβ1, TGFβ2, TGFβ3, αSMA, Col1A1, CK19, CK20 and HBV 3.5kb RNA expression levels were estimated using the ΔΔCt method with normalization to mouse GAPDH RNA [84]. The PCR primers used were 5’-AAGATGGAAGGGCATGAGAG-3’ (mouse FoxA1 sense primer), 5’-CCAGGCCGGAGTTCA-3’ (mouse FoxA1 antisense primer), 5’-GGCCAGCGAGTTAAAGTAT-3’ (mouse FoxA2 sense primer), 5’-TGTTGCTCACGGAAGAGTAG-3’ (mouse FoxA2 antisense primer), 5’-ATGACCTGGCCGAGTGGA-3’ (mouse FoxA3 sense primer), 5’-ATGGTGGGCACAGGATTCAC-3’ (mouse FoxA3 antisense primer), 5’-GCTGCAATGGCTATGGTAGGA-3’ (mouse FoxO1 sense primer), 5’-GTCACAGTCCAAGCGCTCAAT-3’ (mouse FoxO1 antisense primer), 5’-CATCTTCTCAAAATTCGAGTGACAA-3’ (mouse TNFα sense primer), 5’-TGGGAGTAGACAAGGTACAACCC-3’ (mouse TNFα antisense primer) [85], 5’-GAAACTTCATTCAAACCCGGCCCA-3’ (mouse 2OAS sense primer), 5’-CCGGAAGCCTTCAGCAATGTCAAA-3’ (mouse 2OAS antisense primer), 5’-CTCTGCAAGAGACTTCCATCCAGT-3’ (mouse IL6 sense primer), 5’-GAAGTAGGGAAGGCCGTGG-3’ (mouse IL6 antisense primer), 5’-ATTCCTGGCGTTACCTTGG-3’ (mouse TGFβ1 sense primer), 5’-CCTGTATTCCGTCTCCTTGG-3’ (mouse TGFβ1 antisense primer), 5’-GAGCGGAGCGACGAGGAG-3’ (mouse TGFβ2 sense primer), 5’-TGTAGAAAGTGGGCGGGATGG-3’ (mouse TGFβ2 antisense primer), 5’-ATGGTGGTGAAGTCGTGTAAG-3’ (mouse TGFβ3 sense primer), 5’-GTGAGGTCTGTCGCTTTGG-3’ (mouse TGFβ3 antisense primer), 5’-GTCCCAGACATCAGGGAGTAA-3’ (mouse αSMA sense primer), 5’-TCGGATACTTCAGCGTCAGGA-3’ (mouse αSMA antisense primer), 5’-GCTCCTCTTAGGGGCCACT-3’ (mouse Col1A1 sense primer), 5’-CCACGTCTCACCATTGGGG-3’ (mouse Col1A1 antisense primer), 5’-ACTTGCGCGACAAGATTC-3’ (mouse CK19 sense primer), 5’-AACTTGGTTCTGAAGTCATCTGC-3’ (mouse CK19 antisense primer), 5’-GCACAGATTAAAGAGCTGCAAA-3’ (mouse CK20 sense primer), 5’-GTCCTCTGCAGCCAGCTTAG-3’ (mouse CK20 antisense primer), 5’-GCCCCTATCCTATCAACACTTCCGG-3’ (HBV 3.5kb RNA sense primer, coordinates 2311 to 2335), 5’-TTCGTCTGCGAGGCGAGGGA-3’ (HBV 3.5kb RNA antisense primer, coordinates 2401 to 2382), 5’-TCTGGAAAGCTGTGGCGTG-3’ (mouse GAPDH sense primer), and 5’-CCAGTGAGCTTCCCGTTCAG-3’ (mouse GAPDH antisense primer) [46], respectively.

### Serum HBV antigen and liver histology

HBeAg analysis was performed using 2 μl of mouse serum and the HBe enzyme linked immunosorbent assay as described by the manufacturer (Epitope Diagnostics). The level of antigen was determined in the linear range of the assay.

Liver tissue samples were fixed in sodium phosphate-buffered formalin (Fisher Scientific), embedded in paraffin, sectioned (5 μm), and stained with hematoxylin and eosin (H&E) or trichrome (TC) (Electron Microscopy Sciences). Immunohistochemical detection of HBcAg in paraffin-embedded mouse liver sections was performed using a polyclonal rabbit anti-HBcAg primary antiserum (Dako) and a horseradish peroxidase-conjugated goat anti-rabbit immunoglobulin G F(ab’)2 fragment secondary antiserum (Sigma) [21]. The antibody coated slides were subsequently incubated with 3,3’-diaminobenzidine (Sigma) and counterstained with Mayer’s hematoxylin (Sigma).

### DNA methylation analysis

Bisulfite treatment of protein-free genomic DNA for methylation analysis was performed using the EZ DNA Methylation-Lightning Kit (D5030; Zymo Research, Inc., Irvine, CA, USA) according to the manufacturer’s instructions. The 99 CpG sequences in the HBV DNA genome were targeted using PCR amplification of the bisulfite-treated DNA, followed by sequencing of the amplicons on an Illumina MiSeq instrument. Preparation of DNA for high-throughput amplicon sequencing was performed in two PCR steps in a protocol termed “targeted amplicon sequencing (TAS)” as described previously [86, 87]. Thirteen HBV primer pairs targeting 95 of the 99 CpG sites were used. The primer pairs targeting the bisulfite converted HBV DNA were (i) 5’-ACACTGACGACATGGTTCTACACAATACCTAAACCTTTACCC-3’ (oligo CS1FP1; HBV nucleotide coordinates 1131–1150) and 5’-TACGGTAGCAGAGACTTGGTCTGTTTTAGTTAGTGGGGGT-3’ (oligo CS2RP1; HBV coordinates 1214–1197), (ii) 5’-ACACTGACGACATGGTTCTACACAAACTTTCACTTTCTC-3’ (oligo CS1FP1a; HBV coordinates 1086–1102) and 5’-TACGGTAGCAGAGACTTGGTCTGTTGATGGTTTATGATTAA (oligo CS2RP1a; HBV coordinates 1233–1215), (iii) 5’-ACACTGACGACATGGTTCTACACCCCCACTAACTAAAAC-3’ (oligo CS1FP2; HBV nucleotide coordinates 1198–1214) and 5’- TACGGTAGCAGAGACTTGGTCTGGGTAATATTTGGTGG-3’ (oligo CS2RP2; HBV nucleotide coordinates 1645–1630), (iv) 5’- ACACTGACGACATGGTTCTACATTAAACTCTCAACAATATCA-3’ (oligo CS1FP3; HBV nucleotide coordinates 1668–1687) and 5’-TACGGTAGCAGAGACTTGGTCTAAGTTATTTAAGGTATAGTTTG-3’ (oligoCS2RP3; HBV nucleotide coordinates 1896–1875), (v) 5’-ACACTGACGACATGGTTCTACAGTGGTTTTGGGGTATGG-3’ (oligo CS1FP4; HBV nucleotide coordinates 1890–1906) and 5’- TACGGTAGCAGAGACTTGGTCTCAAATTAACACCCACCC-3’ (oligo CS2RP4; HBV nucleotide coordinates 2130–2114), (vi) 5’-ACACTGACGACATGGTTCTACAGGGTGGGTGTTAATTTG-3’ (oligo CS1FP5; HBV nucleotide coordinates 2114–2130) and 5’-TACGGTAGCAGAGACTTGGTCTCCAAAAAATACTAACATTAAAAT-3’ (oligo CS2RP5; HBV nucleotide coordinates 2464–2442), (vii) 5’- ACACTGACGACATGGTTCTACAAGTTATAGAGTATTTGGTGT-3’ (oligo CS1FP5a; HBV nucleotide coordinates 2244–2263) and 5’-TACGGTAGCAGAGACTTGGTCTCCCAATAAAATTCCCCA-3’ (oligo CS2RP5a; HBV nucleotide coordinates 2491–2475), (viii) 5’- ACACTGACGACATGGTTCTACAGATTGTAATTGATTATGTTTG -3’ (oligo CS1FP6; HBV nucleotide coordinates 2625–2645) and 5’- TACGGTAGCAGAGACTTGGTCTCCATACTATAAATCTTATTCCC-3’ (oligo CS2RP6; HBV nucleotide coordinates 2832–2853), (ix) 5’-ACACTGACGACATGGTTCTACAGGGAATAAGATTTATAGTATGG (oligo CS1FP7;HBV nucleotide coordinates 2832–2853) and 5’-TACGGTAGCAGAGACTTGGTCTTAAACCTAAAAACTCCACC-3’ (oligo CS2RP7; HBV nucleotide coordinates 3061–3043, (x) 5’-ACACTGACGACATGGTTCTACAAATCAAAAAAACAACCTACC-3’ (oligo CS1FP8; HBV nucleotide coordinates 3115–3134) and 5’-TACGGTAGCAGAGACTTGGTCTGTGAGTGATTGGAGGT-3’ (oligo CS2RP8; HBV nucleotide coordinates 340–325), (xi) 5’-ACACTGACGACATGGTTCTACAACCTCCAATCACTCAC-3’ (oligo CS1FP9; HBV nucleotide coordinates 325–340) and 5’-TACGGTAGCAGAGACTTGGTCTGTTAAATAGTGGGGGAAAG-3’ (oligo CS2RP9; HBV nucleotide coordinates 730–712), (xii) 5’-ACACTGACGACATGGTTCTACAGGATGATGTGGTATTGG-3’ (oligo CS1FP10; HBV nucleotide coordinates 743–759) and 5’-TACGGTAGCAGAGACTTGGTCTCAAAACCCAAAAAACCCAC-3’ (oligo CS2RP10; HBV nucleotide coordinates 1020–1002), and (xiii) 5’- ACACTGACGACATGGTTCTACATTGATGTTTTTGTATGTA-3’ (oligo CS1FP11; HBV nucleotide coordinates 1053–1070) and 5’-TACGGTAGCAGAGACTTGGTCTATAACCAAACCCCAACC-3’ (oligo CS2RP11; HBV nucleotide coordinates 1222–1206). These primers contained linker sequences (underlined) at the 5’ ends of the oligonucleotides, termed common sequences (CS1 and CS2). These PCR reactions were performed using ZymoTaq PreMix according to the manufacturer’s instructions (Zymo Research). PCR amplification involved 10 minutes denaturation at 95°C, followed by 40 cycles of 95°C for 30 s, 50°C for 40 s, 72°C for 60 s. Finally, a seven 7 minute incubation at 72°C was performed. The HBV amplicons were subsequently subjected to a second stage PCR reaction (AccuPrime SuperMix II, Life Technologies), used to incorporate unique barcodes and sequencing adapters. The PCR primers used were 5’- AATGATACGGCGACCACCGAGATCTACACTGACGACATGGTTCTACA-3’ (oligo PE1CS1) and 5’-CAAGCAGAAGACGGCATACGAGAT**NNNNNNNNNN**TACGGTAGCAGAGACTTGGTCT-3’ (oligo PE2BCCS2). The unique 10 base sample-specific barcodes are indicated with bold Ns and represent a subset of 384 unique sequences (Fluidigm). PCR amplification involved 5 minutes denaturation at 95°C, followed by 8 cycles of 95°C for 30 s, 60°C for 30 s, 68°C for 30 s. Finally, a seven minute incubation at 68°C was performed.

Pooled libraries were sequenced using an Illumina MiSeq instrument and data were analyzed using the Casava1.8 pipeline. Sequencing was performed using MiSeq V2 chemistry, with paired-end 2x250 base reads, employing Fluidigm custom sequencing primers. Raw paired-end sequence data were merged without trimming using the software package PEAR [88]. Merged, trimmed reads were sub-sampled to 2,500 sequences per sample, and mapped to the HBV reference DNA sequence [89]. Greater than 99.9% of reads mapped to the reference sequence. Unmethylated CpG sites were converted to UpG (TpG upon PCR amplification) by bisulfite treatment and the percent methylation at any nucleotide coordinate was determined by the ratio of C residues (derived from 5-methylcytosine) to C+T residues (derived from 5-methylcytosine plus cytosine, respectively) at any position within the HBV genomic DNA sequence. In addition, the methylation status of each read was assessed individually by counting the number of methylated CpG sites observed within that read. The distribution of methylation levels per read allows for the characterization of the cellular heterogeneity in the methylation status at each amplicon.
